# Effective Visual Working Memory Capacity: An Emergent Effect from the Neural Dynamics in an Attractor Network

**DOI:** 10.1371/journal.pone.0042719

**Published:** 2012-08-29

**Authors:** Laura Dempere-Marco, David P. Melcher, Gustavo Deco

**Affiliations:** 1 Department of Information and Communication Technologies, Center for Brain and Cognition, Universitat Pompeu Fabra, Barcelona, Spain; 2 Department of Psychology, Center for Mind/Brain Sciences, University of Trento, Rovereto, Italy; 3 Institució Catalana de Recerca i Estudis Avançats, Barcelona, Spain; Max Planck Institute for Human Cognitive and Brain Sciences, Germany

## Abstract

The study of working memory capacity is of outmost importance in cognitive psychology as working memory is at the basis of general cognitive function. Although the working memory capacity limit has been thoroughly studied, its origin still remains a matter of strong debate. Only recently has the role of visual saliency in modulating working memory storage capacity been assessed experimentally and proved to provide valuable insights into working memory function. In the computational arena, attractor networks have successfully accounted for psychophysical and neurophysiological data in numerous working memory tasks given their ability to produce a sustained elevated firing rate during a delay period. Here we investigate the mechanisms underlying working memory capacity by means of a biophysically-realistic attractor network with spiking neurons while accounting for two recent experimental observations: 1) the presence of a visually salient item reduces the number of items that can be held in working memory, and 2) visually salient items are commonly kept in memory at the cost of not keeping as many non-salient items.

Our model suggests that working memory capacity is determined by two fundamental processes: encoding of visual items into working memory and maintenance of the encoded items upon their removal from the visual display. While maintenance critically depends on the constraints that lateral inhibition imposes to the mnemonic activity, encoding is limited by the ability of the stimulated neural assemblies to reach a sufficiently high level of excitation, a process governed by the dynamics of competition and cooperation among neuronal pools. Encoding is therefore contingent upon the visual working memory task and has led us to introduce the concept of effective working memory capacity (*e*WMC) in contrast to the maximal upper capacity limit only reached under ideal conditions.

## Introduction

### General background and motivation

Working memory (WM) provides temporary storage and manipulation of the information necessary for accomplishing complex cognitive tasks, and, as stated by Baddeley [1] it stands at the crossroads between memory, attention, and perception. Its study and understanding is, thus, central in cognitive psychology. Baddeley proposed a WM model [2] consisting of three subsystems: the “phonological loop” dealing with verbal information, the “visuospatial sketchpad” concerning visual information, and the “central executive system” allowing the manipulation and control of information in WM. It is also known that the human visual system is divided into object and spatial information processing pathways, and such distinction has also been found in WM systems [3]. Throughout this study, we will focus on the study of visual object working memory and its capacity limits.

Since WM capacity is a basic aspect of cognition, capacity limitations have been well studied in humans, thus leading to a wide psychophysical literature (e.g. see [4] for a review). Many studies support the view that visual WM shows strict upper limits of around 3–4 items [4], although the lower limit in capacity varies depending on the participants and task parameters [5]–[7]. Interestingly, the study of visual WM capacity and accuracy have recently attracted renewed attention. For instance, Todd and Marois [8] found that performance declined with increased set size in a visuospatial WM task that consisted of deciding after a 1200 ms retention interval whether a particular coloured disk was previously shown in a display containing one to eight coloured discs. They also found that the number of objects encoded at each set size, estimated using Cowan's 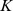 equation [4], increased up to a set size 3 or 4 and levelled off thereafter. Zhang and Luck [9] also showed how performance drops with set size, slowly from 1 to 3 and then suddenly at set size 6, in a colour recall task that also assessed the precision with which the colour was retained in memory.

There are currently a number of theories regarding the underlying mechanisms that yield capacity limits. The main two competing models include “fixed capacity models” (or slot models) [9], and dynamic allocation models (or resource models) [5]. In fixed capacity models, all items are recalled with equal precision up to the limit (3–4 items), and it is predicted that no information is stored about items beyond this limit. In contrast, in dynamic allocation models, the limited resources are shared out between items but not necessarily equally. Importantly, this model predicts that all of the items get allocated some resources. It is worth noting that visual saliency provides a benchmark to compare the predictions of these competing theories allowing the dynamical allocation of resources to be probed.

### The role of attention and saliency in visual working memory

Visual saliency has been extensively studied in the context of visual attention. One of the most influential theories of visual attention was Treisman and Gelade's Feature Integration Theory [10], which is closely related to saliency maps. The saliency map concept was originally introduced by Koch and Ullman [11] and has inspired many computational models [11], [12]. The original notion refers to the conspicuousness of a particular location on the basis of its bottom-up distinctiveness relative to that of other locations in the scene. The neurophysiological substrate of saliency maps is still a matter of investigation. Moreover, it is unclear whether saliency maps arise in a particular brain area or else are distributed among different areas including superior colliculus (SC) [13], frontal eye field (FEF) [14], posterior parietal cortex (PPC) [15], or the primary visual cortex (V1) [16]. In computational models, the saliency map is often defined as a topographical map that combines information from several elementary feature maps into a global measure of conspicuousness [11].

There are few studies investigating how visual saliency affects cognitive functions other than attention, in particular, how visual saliency affects WM. Furthermore, seemingly contradictory conclusions are derived from those studies which have addressed this issue. On one hand, Fine and Minnery [17] find that the ability to recall an object's spatial location is positively correlated with the object's saliency in the visual scene, and that this is not a consequence of biasing overt attention. On the other hand, Berg and Itti [18] found that the computed saliency of object patches had no significant correlation with subjects' recall rates in a WM task. Nonetheless, overt attention surrogates such as eye position and fixation time on an object appeared to strongly facilitate recall in such task. Since both tasks targeted different WM systems, and as pointed out by Fine and Minnery [17], an intriguing possibility is that saliency affects the encoding of spatial memories to a larger extent than memories related solely to object identity.

Interestingly, Bays and Husain [5] also pointed out that eye movements do play a significant role when assessing performance in WM tasks. In particular, they suggest that when eye movements take place, the item at the target location obtains more resources and is therefore recalled with greater precision. They manipulated visual attention in location-judgment and orientation judgement tasks by flashing an item prior to blanking the screen. When the flashed item was subsequently probed, discrimination precision was significantly higher than for nonflashed items, thus suggesting that certain visual information is given priority for storage in WM. Their predictions of the probability of correct responses also show a decrease in performance with increased set size although, for large set sizes, the model would predict that a large number of items (larger than 5
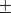
2) could be stored in memory. Zhang and Luck [9] also reported how cueing the position that would be later assessed implied a significant increase in performance (i.e. as assessed from the probability that a particular item was present in memory) for valid cues while rendering lower performances for neutral cues and much lower performances for invalid cues. Similar results were encountered when shape instead of colour recall was considered. However, in this task, the stimulation period was increased to ensure appropriate coding during the display of stimuli.

Finally, in a recent study Melcher and Piazza [19] show that saliency determines the capacity limit in various tasks. To this end, they manipulated bottom-up saliency (visual contrast) and top-down saliency (task relevance) in enumeration and visual memory tasks. As one item became increasingly salient, memory performance for all other less-salient items was decreased. Overall, the pattern of results suggests that our abilities to remember small groups of stimuli are grounded in an attentional priority or saliency map which represents the location of relevant items and that visual WM capacity is influenced by changes in the relative saliency of the items. Memory for the most salient item remained high, independent of increased set size, while performance for the non-salient item dropped suddenly with set size. Similar results were found in trials in which one item was more salient because it was presented at the saccade target location. Thus, both bottom-up and top-down saliency influenced visual WM in similar ways. Furthermore, they observed that saliency manipulation decreased the overall capacity estimate (Cowan's 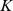 estimate). In particular, by varying the difference in contrast between the salient item and the remaining ones, it was shown that memory for the salient item remained relatively constant while performance for the non-salient items decreased as the relative saliency difference increased, thus leading to an overall reduction in behavioural performance. These experiments contribute a series of new experimental results which previous computational models did not address.

In this work, we propose a biophysically-realistic computational model that provides an explanation of how visual saliency may shape WM function, thus rendering an effective WM capacity (*e*WMC) in contrast to a maximal upper capacity limit only found under particular conditions. We will pay special attention to the idea that the relative saliency of items depends on the competition between the various items in a visual display and, in the limit, if one item is particularly salient compared to the other items, it can become the only one strongly represented in the map [15].

### Neurophysiological correlates of visual working memory

In the neurophysiological arena, selectively enhanced activity throughout the delay period of delayed match-to-sample tasks has been traditionally regarded as a neural correlate of WM function and has been found in different brain areas, such as prefrontal cortex (PFC) (e.g. [20], [21]), inferotemporal cortex (IT) (e.g. [22]), and intraparietal sulcus (IPS) in posterior parietal cortex (PPC) (e.g. [8]). These neurophysiological studies have been mostly concerned with the storage of single items in WM and have inspired multiple computational models of WM, including ours, as will be discussed in the next section.

However, the mechanisms underlying the encoding and maintenance of multiple items in WM have not been clearly identified and several candidates have been suggested, namely 1) sustained neural activation, 2) neural oscillations, or 3) patterns of synaptic strength. Experimental evidence for the first mechanism is available from experiments dealing with single items, such as those previously reported, whereas evidence for a relevant role of oscillations or patterns of synaptic strength in WM mainly comes from EEG studies in which power increases in different frequency bands and different brain locations have been observed [23]–[25]. Nonetheless, it is worth noting that the role of oscillations in determining WM capacity is still not well understood and such observations are not exempt of controversy with different studies suggesting increases/decreases in power with WM load in different frequency bands (e.g. [23]–[25]). Furthermore, the locus (or loci) of WM function leading to capacity limits have not been fully established. Although PFC is a clear candidate and has been the object of study in neurophysiological studies such as that by Warden and Miller [26], other studies dealing with visuospatial WM tend to localise them in the parietal cortex (e.g. [27]).

There have been very few studies which examine neural activity for multiple, simultaneously presented items which look at the temporal dynamics of selecting and maintaining stimulus representations. Only very recently, a neurophysiological investigation has addressed the study of the neural substrates of WM capacity limits [28]. In this study, single cell activity from parietal and frontal cortex of two adult monkeys was simultaneously recorded while the animals were engaged in a change detection task in which multiple visual items were concurrently shown in a display. The reported results suggest that capacity limits in visual WM arise from competition for encoding within neuronal pools. It is also suggested that information about multiple objects multiplexes in PFC, following the observation that the response of the neurons depends on the presence of the various items in the display, a result compatible with previous experimental evidence by Warden and Miller [26].

### Computational models of working memory

A number of computational models have been proposed to account for WM. The different models attempt to account for one of the three feasible neural mechanisms that may underlie WM, as previously pointed out. By and large, sustained neural activation during the delay period is the mechanism that has received the most attention from the modelling community and attractor networks have proven to be successful to account for this phenomenon. Attractor networks are networks of neurons endowed with excitatory connections that may settle into (self-sustained) stable patterns of firing. Among attractor networks one can distinguish between discrete attractor networks (e.g. [29], [30]) and continuous attractor networks (e.g. [31]). Although they share a common architecture, in continuous attractor networks recurrent collateral connections between neurons reflect the distance between neurons in a given state space that varies continuously along a physical dimension such as orientation, spatial position, etc. Consistent with neurophysiological recordings, these models are able to reproduce two different types of collective activity: spontaneous rates or selectively enhanced activity during a delay period. Such collective activity impose certain constraints on the model, namely, local inhibition is necessary in order to enable stable states with spontaneous rates, and an average synaptic long-term potentiation (LTP) in specific populations is required to give rise to local attractors with sustained high firing rates during the delay period. Networks of integrate-and-fire neurons are commonly used in this arena since they offer biological plausibility.

Alternatively, Lisman and Idiart [32] propose a different mechanism based on the idea that firing may be sustained by an increase of the membrane excitability that is refreshed on each cycle of a network oscillation. The neuromodulators acetylcholine and serotonin, released during brain oscillations, induce an afterdepolarisation which results in a transient increase in excitability of the neurons. Thus, the model consists of a network of excitatory and inhibitory cells in which pyramidal cells make converging excitatory synapses onto an interneuron which, in turn, produces feedback inhibition of the excitatory cells. The excitatory cells receive a brief stimulation (i.e. informational input modelled as a suprathreshold excitatory input) and a subthreshold low-frequency oscillation. This mechanism enables several sequential memories to be stored and the number of short-term memories that can be stored is limited by the number of high-frequency subcycles that fit within each low-frequency cycle.

Finally, Mongillo et al. [33], although still within the attractor network framework, introduce synaptic dynamics enabling the storage of memories without requiring elevated firing rates during the delay period. In particular, they propose that WM is sustained by calcium-mediated synaptic facilitation, such that presynaptic residual calcium acts as a buffer that is loaded, refreshed and read out by spiking activity. This, in fact, addresses the experimental observation that modest activity increases (or even disappearance of activity) during the delay period may occur [34]–[36], and therefore, WM might not reside exclusively in spiking activity. Similarly to Lisman and Idiart [32], a periodic stimulation may be at the basis of the maintenance of items in WM leading to the emission of a population spike that reactivates the stored memory. However, it is also possible to autonomously induce such reactivation in this model by increasing the overall background input received by the network which, for sufficiently large values, may even lead to a sustained high firing rate during the delay period. Only in such limit it shows persistent activity and can, otherwise, show oscillations.

It is worth pointing out that the hypothesis that selective populations of excitatory neurons are formed is common to most computational studies (including ours), in particular to all the studies in which persistent activity is modelled (e.g. [30] and [27]). Whether persistent activity is invoked or not does not pose a fundamental difference, and thus, attractor networks can also be used to model oscillations. As previously discussed, Mongillo et al. [33] can reproduce both persistent and oscillatory behaviour by adding short-term synaptic facilitation and adaptation to an attractor network. However, they also impose that distinct populations of selective excitatory neurons are formed. Similarly, Lundqvist et al. [25], also require some sort of synaptic potentiation method (i.e. they use long-term potentiation as well but suggest that fast hebbian learning could be considered too) in order to create distinct populations that store different items.

Interestingly, only a few authors have attempted to model multi-item WM (e.g. [27], [32], [37], [38]), and most of these studies address this issue by dealing with sequential stimulation of the memory set rather than simultaneous stimulation. This is the case of the study by Amit et al. [38], who proposed an extended mean field model of multi-item WM that, based on the approach by Brunel and Wang [30], attempts to encapsulate finite size-noise in mean field theory. It thereby allows fast computational simulations of an otherwise largely time consuming simulation protocol. They reproduce the experimental results of a sequential test, inspired by the distractor experiments of Miller et al. [39] (and described in detail in Yakovlev et al. [40]), whereby macaque monkeys were trained to recognise the repetition of one of the images already seen in a sequence of random length. Both the computational [38] and experimental results [40] suggest that up to 6–7 items may be held in WM simultaneously. These results are in contrast to those reported by previous models such as Brunel and Wang's [30] which indicate that sample specific persistent activity may be disrupted when distractors are shown during the delay period of a delayed-response task.

Macoveanu et al. [37] also propose a model of visuospatial WM that is able to simultaneously store multiple items. To this end, they built on previous models that successfuly implemented one item WM and implemented putative cellular changes that occur during development, such as synaptic remodeling, in order to capture the ability to store multiple items in WM. Of particular interest to our study is the work by Edin et al. [27], in which a continuous attractor network is used to model visuospatial WM and the mechanisms underlying WM capacity are analysed in depth. The computational model is, in fact, similar to that in [37]. The authors show that there exists an upper boundary to the capacity limit arising from lateral inhibition in parietal cortex but mnemonic capacity can be boosted by excitatory prefrontal input, thus accounting for interindividuals variability. The capacity limit, however, is investigated by following a mean field analysis which restricts the study to the stationary state. Therefore, no attention is paid to the transient period during which stimuli must be loaded into memory while, in fact, the dynamics of the system during this period may determine whether a given stimulus is successfully encoded in WM and can then be kept throughout the delay period. In terms of the attractor picture, this implies that the dynamics of the system during the transient period will be responsible for which basin of attraction the system enters, and thus, imposes some restrictions to how many objects can be possibly held in WM. In this study, we will address this issue by manipulating not only the size of the memory set but also the relationship between the items in such set in terms of saliency.

## Results

**Figure 1:**
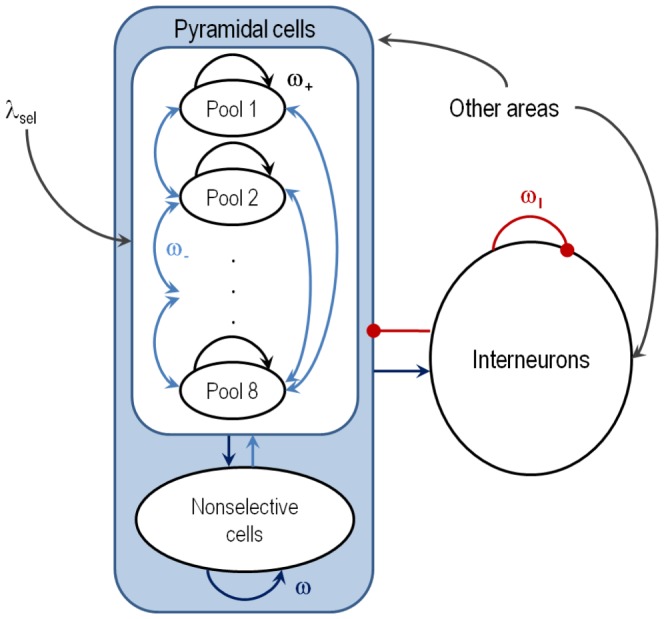
Architecture of the cortical network model. The population of excitatory neurons is subdivided in non-overlapping populations selective to 8 different stimuli. Black and blue arrows within pyramidal cells: NMDA and AMPA-mediated recurrent excitatory connections. Black arrows from other areas: AMPA-mediated external excitatory connections. Red circle-headed arrows: GABA-mediated inhibitory connections. There are three possible synaptic strengths for recurrent excitatory connections: potentiated (by a relative factor 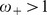, black arrows), depressed (by a relative factor 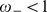, light blue arrows), and unchanged (baseline level 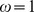, dark blue arrows). The weight 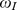 denotes the strength of inhibitory-to-excitatory and inhibitory-to-inhibitory connections. The dots stand for the missing 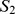, …, 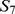 populations and their corresponding connections.

To understand the neural basis of WM capacity, a biophysically-realistic computational model of visual object WM in PFC is presented. The proposed model is based on that introduced by Brunel and Wang [30], which accounts for object WM when a single pool is stimulated at once. In this work, we have, however, considered the simultaneous stimulation of multiple items in a visual display with different visual saliencies. To this end, a network structured into 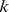 statistically homogeneous neural populations has been considered. In particular, the statistical properties of the synaptic currents and the connection strengths are identical for all the cells from the same population. There is one population of inhibitory cells and one population of excitatory cells, which is partitioned into 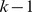 subpopulations. 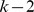 of these represent the ensemble of excitatory neurons selective to each object while the (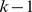)th subpopulation includes the remaining nonselective cells. Recurrent connections between cells from the same selective subpopulation are potentiated by a factor 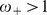 with respect to the baseline connectivity level, while connections between cells from different selective subpopulations are weakened by a factor 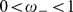. This follows the hypothesis of Hebbian plasticity, i.e. synaptic efficacies are modified by neural activity following a training process. Since it has long been established, based on experimental observations, that neuronal activity affects synaptic strength through long-term potentiation (LTP) and long-term depression (LTD), we assume that our network has been structured through repeated presentations of 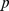 different stimuli at random sequences. The strength of inhibitory-to-excitatory connections and inhibitory-to-inhibitory connections is denoted by the weight 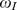. In this study, we have considered a network which has learned 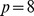 stimuli (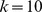). As in Brunel and Wang [30], the proposed cortical model consists of a fully connected recurrent network of integrate-and-fire neurons with realistic synaptic excitation. A schematic representation of the network architecture is shown in Fig. 1. The synaptic coupling strengths of the proposed model were calibrated using a mean field analysis in order to obtain desired levels of spontaneous activity and comply with the main conclusions derived from the experimental results published in the literature.

### Mean field analysis

Although simulating populations of individual neurons is necessary to reproduce realistic neuronal dynamics, in order to understand the underlying attractor and dynamical structures governing the dynamics of the neural populations, a simpler model encapsulating the average activity of these populations can be used. This is accomplished by considering a mean field approximation. The details of this approximation can be found in the original publication [30] and in (Text S1). The large number of integration variables characteristic of spiking models is reduced to one for each neural population in the mean field approach, this allows faster calculations and the parameter space can, in fact, be exhaustively scanned. By solving the mean field equation for a set of initial conditions, one obtains the average firing rate of each pool when the system has settled into a stationary state, which corresponds to the attractors (i.e. stable states) of the system. The initial conditions in this study correspond to the initial firing rate of each neural population.

Since we aim to account for the main phenomena described in the literature regarding WM capacity, namely: a) in absence of saliency effects, recall performance drops with set size, slowly from 1 to 3–4 but showing a large drop off for larger sizes (e.g. [5], [8], [19]), b) preferential storage in memory of salient items (e.g. [5], [9], [19]), and c) reduction in the total number of items that can be stored in WM in the presence of visually salient items (e.g. [19]), a dynamical regime compatible with such results must be identified. To this end, using the mean field approximation of the model, we analysed the network behaviour as a function of the synaptic weights 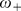 and 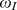.

**Figure 2:**
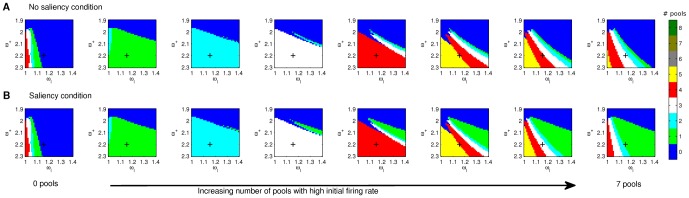
Mean field analysis of the model. Mean field analysis of the model assessing the dependence of the network behaviour on the potentiated synaptic strength (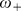) and the inhibitory synaptic strength (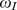), for different initial conditions. **A** The initial firing rate conditions for pools showing high firing rates are derived from a Gaussian distribution with mean 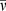 = 40 Hz and standard deviation 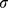 = 0.01 Hz. The firing rates determining the initial conditions of pools in spontaneous states are obtained from randomly sampling a Gaussian distribution with mean 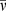 = 3 Hz and standard deviation 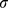 = 0.01 Hz. The colour code indicates the number of pools which settle on stable states showing persistently high firing rates (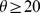 Hz) during the delay period when no further stimulation is provided. **B** Identical initial conditions as in **A** but one of the pools showing an initially high firing rate of 65 Hz. From left to right an increasing number of pools had high initial firing rates. Note that as a consequence of considering a hard boundary (i.e. 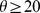 Hz, used in subsequent studies) for values 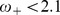 some apparent discontinuities may appear for increasing 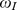 values, which in fact correspond to stable states with persistent firing rates just below the threshold. However, this does not occur in the region where our working point is located (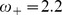, 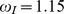).

In particular, we assessed the number of pools that simultaneously showed persistent elevated activity (i.e. firing rate above a threshold set to 20 Hz) in the stationary state as these were considered the neural correlates of item maintenance in WM in contrast to those pools showing steady states at spontaneous levels of activity (i.e. 1–3 Hz). A number of initial conditions were probed and, for those illustrated in Fig. 2, we verified that only those pools that received stimulation did settle in stable states with persistently high activity. The results obtained for these initial conditions are compatible with the conclusions from the analytical study by Edin et al. [27], who offered a mechanistic explanation for the existence of an upper boundary capacity limit grounded on lateral inhibition. As discussed by Edin et al. [27], Fig. 2 also shows that, the farther above capacity the load is, the fewer items are subsequently maintained in WM (i.e. less pools in persistent states with elevated firing rates are found).

Thus, a working point 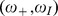 was selected such that the main experimental findings reported in the literature could be reproduced. For this working point, a set of 1000 different initial conditions randomly selected were probed to provide further evidence for the existence of an upper boundary capacity limit, which was again found to be of 4 items. This point is indicated in Fig. 2 by means of a black cross and, as can be observed, it is found that: a) an upper capacity limit around 4 items is encountered (in agreement with the results shown in [8] and as predicted by [27]), b) the number of items that can be held in memory in the presence of salient items is reduced if compared with the situation when no saliency effects are present, and c) although not shown in this graph, salient items are preferentially stored in memory (i.e. the visually salient item is always among those settling in a steady state with high firing rate). These predictions must be nevertheless verified with a full spiking network model since the mean field picture constitutes only an approximation that may in fact be weak in the proximity of bifurcation points [30].

It is worth noting that, although a large variety of experimental designs can be found in the literature (e.g. different stimulation time as a consequence of the nature of the stimuli and the requirements of the task), the working point of the proposed model is not adjusted to specifically reproduce any particular experiment but rather to encapsulate general trends that arise from the different experiments. As shown in Table 1, the selected working point corresponds to 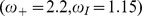; however, the results shown in Fig. 2 suggest that similar results should be expected over a wide range in the 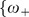, 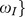 parameter space.

**Table 1:** Parameters of the integrare-and-fire simulations.

PARAMETER	VALUE
**Network parameters**	
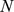 : number of neurons in the network	10000
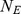 : number of excitatory neurons	0.8 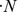
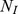 : number of inhibitory neurons	0.2 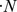
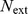 : number of external neurons	800
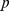 : number of selective populations	8
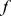 : fraction of excitatory cells in each selective population	0.1
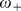 : relative strength of single potentiated synapses	2.2
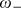 : relative strength of single depressed synapses	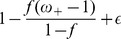
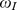 : relative strength of inhibitory synapses	1.15
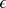 : additive term to the homeostatic condition	0.02
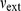 : spike rate at external synapse	2.4 kHz
**Neuronal parameters (excitatory and inhibitory)**	
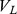 : resting membrane potential	−70 mV
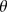 : firing threshold	−50 mV
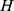 : reset potential	−55 mV
**Neuronal parameters (excitatory)**	
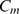 : membrane capacitance	0.5 nF
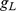 : membrane leak conductance	25 nS
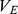 : reversal potencial	0 mV
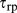 : refractory period	2 ms
**Neuronal parameters (inhibitory)**	
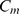 : membrane capacitance	0.2 pF
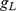 : membrane leak conductance	20 nS
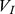 : reversal potencial	−70 mV
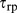 : refractory period	1 ms
**Synaptic parameters (excitatory and inhibitory)**	
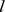 : synaptic latency	0.5 ms
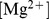 : extracellular magnesium	1 mM
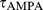 : decay time of AMPA currents	2 ms
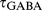 : decay time of GABA currents	10 ms
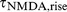 : rise time of NMDA currents	2 ms
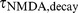 : decay time of NMDA currents	100 ms
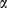 : normalisation factor for NMDA PSCS	0.5 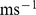
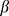 : gain factor in magnesium block	0.062 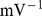
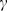 : modulatory factor of magnesium block	3.57 mM
**Synaptic parameters (excitatory)**	
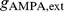 : external AMPA synaptic conductance	2.08 nS
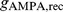 : recurrent AMPA synaptic conductance	104 nS/ 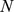
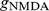 : recurrent NMDA synaptic conductance	327 nS/ 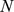
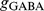 : recurrent GABA synaptic conductance	1250 nS/ 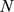
**Synaptic parameters (inhibitory)**	
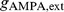 : external AMPA synaptic conductance	1.62 nS
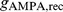 : recurrent AMPA synaptic conductance	81 nS/ 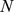
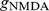 : recurrent NMDA synaptic conductance	258 nS/ 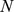
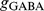 : recurrent GABA synaptic conductance	973 nS/ 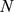

Although the mean field analysis has yielded results that match well some experimental evidence, when assessing the effects of visual saliency on item maintenance in working memory, Fig. 2 shows that these are only particularly noticeable in those cases in which more than four pools present initially high firing rates, that is, above capacity. This is, however, in contrast to the experimental results reported by Melcher and Piazza [19], which suggest that saliency effects are relevant also for set sizes under the capacity limit established in the no saliency condition. It could still be that the network behaviour in the steady state does not fully determine WM capacity and, therefore, the dynamical behaviour of the system during the transient period may also play an important role in yielding such capacity limit. In order to verify this prediction the full spiking network model must be used since it provides detailed information about the complete dynamics of the system and not just about its behaviour in the stationary state.

### Computational model predictions

We performed spiking simulations to analyse the neural basis of WM capacity in a delayed match-to-sample task. This is a task commonly used, and with results broadly reported in the literature, to assess WM capacity. The proposed WM model predicts the firing rates of selective neural ensembles during the delay period of the task. Thus, the model provides a mechanistic explanation of the neural correlates of WM, and we propose that reading out such neural responses is necessary in order to make informed decisions.

Following up on the results of the mean field approach previously presented, it should be noted that the initial conditions used in the mean field approximation are only reached after applying a particular stimulation protocol. The delayed-response protocol considered throughout this study is simulated as follows: (1) the simulation starts with a pre-cue time interval of 1000 ms, during which the network exhibits spontaneous activity, then (2) the stimuli presentation consists of a transient input lasting for 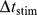 to those cells selective to the shown stimuli, which is implemented by an increase in the input frequency from 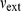 to 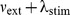, where 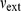 represents the background signal associated with spontaneous activity outside the network and 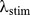 represents the response of the selective neurons in PFC to the visual stimuli displayed in the memory set and corresponds to a few tens of Hz, while other cells are unaffected.

Finally, (3) after the external stimuli are removed, there is a delay period 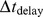. The neural responses during such delay period are regarded as the neural correlates of WM. In those trials which included saliency effects, the effect of saliency was considered by further increasing the input frequency to the salient selective pool to an amount 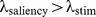 proportional to the saliency of the visual item. Neurophysiological evidence suggesting such modulatory effect was presented by Everling et al. [41].

Since no direct measures matching our simulation protocols are available in the literature, throughout this paper we present the direct firing rate predictions from our model and also an alternative indirect validation of the proposed model by means of behavioural predictions. These are derived from the distributions of the number of items maintained in visual WM, which constitute primary predictions of the model too, and can be subsequently compared to results reported in the literature to assess whether equivalent qualitative trends are reproduced. Moreover, we propose new behavioural predictions that are grounded on the neurodynamical mechanisms described in this work.

The variable selected to characterise behaviour in this study is performance since its use is extensively reported in the literature (e.g. [4], [5], [9], [19]). Unless otherwise stated, the behavioural theoretical performance estimates (described in detail in the Methods section) assume a naïve system -built on top of the proposed network model- which does not develop any strategy to maximise performance, i.e. performance is only estimated on the basis of the neural activity during the delay period. To this end, we have assumed two possible scenarios: 1) the test item is randomly retrieved from the pool of visual stimuli that have been learned by the network (i.e. the test object is one of the eight stimuli encoded by the network) or 2) the test object is randomly selected from the pool of visual stimuli that have been displayed during the stimulation period. In the first case, performance is measured by the proportion of correct responses as derived from both true positive trials (i.e. correct maintenance of an object in the memory set) and true negative trials (i.e. correctly recalling that an item was not present in the memory set) (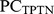) (see Eq. 7 in Methods), whereas in the second scenario performance is measured by the proportion of correct responses derived from true positive trials (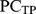) (see Eq. 8 in Methods). It should be considered that provided the selectivity profile of the neurons (i.e. perfectly tuned to the learned items), should an item be correctly encoded and maintained in WM, an error free recognition is postulated. In this way, the probability of correct recognition becomes always one and performance estimates can be obtained from the histograms conveying information about the number of stimuli maintained in WM after stimulation for each memory set. Two different theoretical performance estimates have been considered in order to assess whether the conclusions derived from our study are sensitive to the specific experimental design employed to assess WM capacity. It turns out that both measures provide qualitatively similar results.

**Figure 3:**
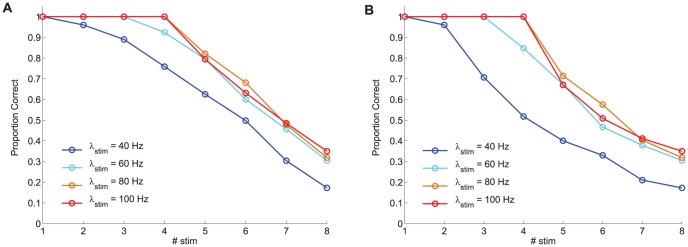
Model-based prediction of performance for different levels of external stimulation. Model-based prediction of performance derived from computational simulations of a change detection task with 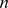 selective neural assemblies (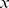 axis) simultaneously stimulated. Performance is calculated by assuming that an item is held in visual WM when its associated selective pool shows a mean persistent activity 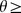
20 Hz during the last 300 ms of the delay period. 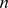 selective pools are stimulated at different amplitude levels 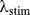 = 40 Hz, 60 Hz, 80 Hz, and 100 Hz. **A** Performance calculated as 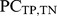 (Eq. 7), and **B** performance calculated as 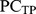 (Eq. 8). For both proposed performance estimates, performance decreases for larger set sizes but improves for larger stimulation amplitudes up to a value (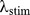 = 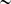
80 Hz) beyond which performance seems to converge.

**Figure 4:**
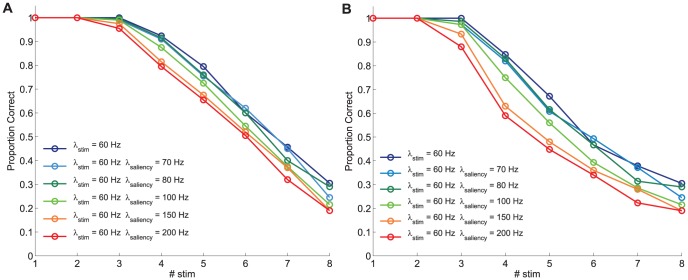
Model-based prediction of performance for different levels of saliency. Model-based prediction of performance derived from computational simulations of a change detection task with 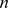 selective neural assemblies (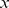 axis) simultaneously stimulated. Performance is calculated by assuming that an item is held in visual WM when its associated selective pool shows a mean persistent activity 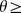
20 Hz during the last 300 ms of the delay period. 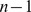 selective pools are stimulated at 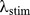 = 60 Hz and the remaining pool receives a higher stimulation 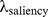 = 60 Hz (no saliency), 70 Hz, 80 Hz, 100 Hz, 150 Hz, and 200 Hz. **A** Performance calculated as 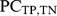 (Eq. 7), and **B** performance calculated as 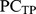 (Eq. 8). For both proposed performance estimates, performance decreases for larger set sizes and for larger saliency levels.

Preliminary simulations were conducted in order to determine an appropriate level of external stimulation, 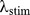, matching the results reported in the literature regarding visual WM capacity limits. In the simulation protocol employed for these preliminary simulations we have used 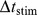 = 500 ms and 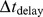 = 3.5 s. Throughout this study, WM capacity has been assessed by counting the number of items that are maintained (i.e. those showing an elevated firing rate, 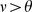 = 20 Hz) during two different intervals within the delay period: 1) the last 300 ms of the delay period, and 2) the last 2 s of the delay period. It is worth pointing out that only subtle differences (i.e. at most 1% of the trials show a different outcome) have been observed, which leads us to conclude that stable memories are encoded and maintained. Fig. 3 shows the model prediction for the proportion of correct responses (PC) for different values of 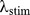 with one hundred trials simulated for each condition. Although similar qualitative trends are found for both performance estimates, it is worth noting that the specific experimental design employed to probe WM capacity should be carefully considered when interpreting the performance results since there exist quantitative changes. For instance, 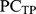 decreases more abruptly for 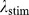 = 40 Hz from set size 3 than 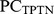 (see Fig. 3), and the detrimental effect of saliency on overall performance is more pronounced also for 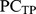 than for 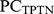 (see Fig. 4).

In agreement with previously published experimental results, and as predicted from our mean field analysis, the visual WM capacity limit upper boundary is found at around 4 items. As can be seen from Fig. 3, for an intensity 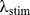 = 80 Hz, the visual WM capacity limit is reached. Indeed, such limit is determined (as thoroughly discussed in Edin et al. [27]) by the competition between the different selective neuronal pools during the delay period, which is largely mediated by inhibition. For intensities 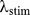 over 80 Hz, all stimulated items also reach a sustained high firing rate and, only upon retrieval of the stimuli, such firing rate activity decays under threshold level for several pools. For intensities 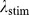 between 60 and 80 Hz, the frequency with which four pools reach, upon stimulation, a high firing rate state during the stimulation period and maintains it throughout the delay period is slightly reduced, thus implying a small reduction in estimated behavioural performances. In contrast to the previous case, substantial differences are observed between the cases with intensities 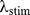 = 40 Hz and 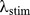 = 80 Hz. Thus, below a certain intensity, around 60 Hz, the predicted performance decreases considerably. As discussed in the next sections, this indicates a failure to appropriately encode the items into WM.

Inspired by the results shown by Melcher and Piazza [19] on the impact of saliency on visual WM capacity, we run several simulation conditions emulating the role of saliency. To this end, several different levels of saliency were considered in the model by varying 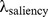 (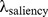 = 70 Hz, 80 Hz, 100 Hz, 150 Hz and 200 Hz), over a baseline condition characterised by an intensity 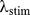 = 60 Hz. The curves in Fig. 4, thus, depict the results from simulations in which all stimulated pools, but the pool responding to the visually salient item, receive a baseline intensity 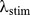, whereas the pool selective to the salient item receives an intensity 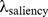. As can be clearly seen from these results, the model predicts that increasing the level of saliency will lead to a decrease in performance as measured by the magnitude PC. Such detrimental effect is nicely graded as a function of visual saliency for both estimates. These results are in agreement with the experimental results reported by Melcher and Piazza [19] and, as discussed next, the model provides a mechanistic explanation that may underlie the observed behavioural results.

**Figure 5:**
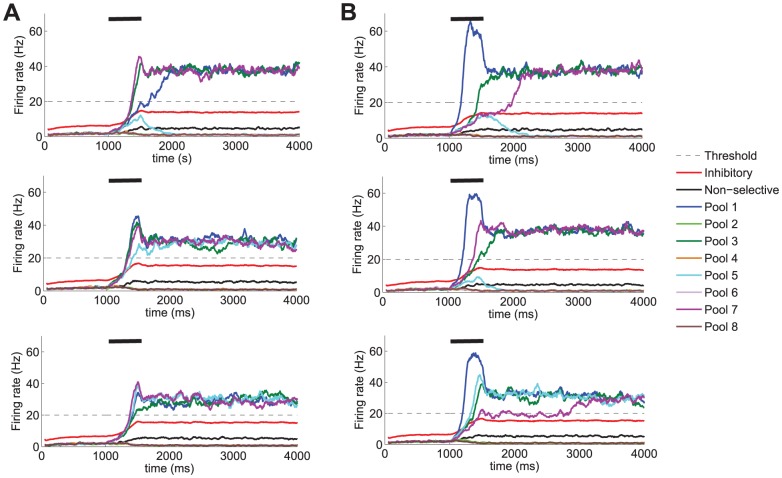
Prediction of firing rates. Results obtained from the network model with the parameters shown in Table 1. The stimulation period during which the external stimulation is administered to the network to emulate the presence of 4 items in the visual display is 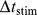 = 500 ms and is depicted by a black segment. **A** 4 pools (pool 1, pool 3, pool 5 and pool 7) receive an external stimulation 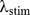 = 60 Hz and **B** the pool selective to the salient item (pool 1) receives an external stimulation 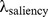 = 100 Hz while the remaining three stimulated pools (pool 3, pool 5 and pool 7) receive only 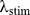 = 60 Hz. As a consequence of the biased competition in the visual saliency condition, the pool selective to the salient item quickly reaches a state of high firing rate during the stimulation period while preventing others from accessing such a state. This leads to fewer items being appropriately encoded into the visual WM system and, thus, would reduce performance in those trials in which the test item is different from the salient one.

**Figure 6:**
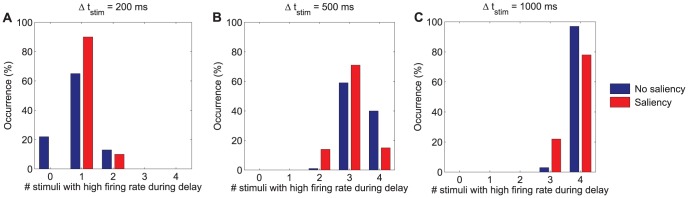
Distribution of number of items maintained in visual WM as a function of the stimulation period. Histograms illustrating the percentage of trials in which multiple items show high activity during the last 300 ms of the delay period for different stimulation periods: **A** 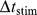 = 200 ms, **B** 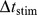 = 500 ms, and **C** 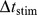 = 1000 ms. The set size of the memory set is 4 in these experiments. Four pools receive an external stimulation 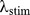 = 60 Hz in the no-saliency condition whereas the pool selective to the salient item receives an external stimulation 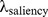 = 100 Hz (while the remaining three stimulated pools receive only 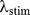 = 60 Hz) in the saliency condition. The network parameters employed in these simulations are those shown in Table 1. The results illustrate that *e*WMC increases when the stimulation period is increased and is reduced in the presence of saliency.

In order to facilitate a detailed analysis of these results and focus the discussion on the neural mechanisms underlying visual WM capacity, a specific case of particular interest will be studied. In particular, the case when 4 pools are simultaneously stimulated. As reviewed in previous sections, a capacity limit of around 4 items is commonly found in psychophysical experiments which according to the results presented in Fig. 3 and Fig. 4 is well reflected by an intensity 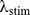 = 60 Hz. In this study, the selective pool responding to the visually salient item in the saliency condition receives an incoming signal of amplitude 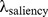 = 100 Hz during the stimulation period 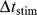. As can be seen from Fig. 5 and Fig. 6B, in most cases, at least three pools show a sustained high firing rate during the delay period. As expected, noise, modelled as a stochastic component of the model, introduces some variability in the dynamics of the system, which in turn implies that the number of items held in memory during the delay period may slightly vary from trial to trial. However, a clear trend is observed in the results showing a considerably larger proportion of trials keeping 4 items in memory in the no-saliency condition than in the saliency condition (see Fig. 6B,C). Upon observation of the dynamical evolution of the firing rates of the different neuronal pools (Fig. 5), it becomes clear that the activity of the inhibitory pool in the condition with no saliency is commonly driven by the collective behaviour of several selective neuronal pools. This possibilitates that several pools take advantage of the recurrent currents associated with potentiated synapses at lower inhibition rates and earlier during the stimulation period. In contrast, in the condition when visual saliency is present, it is the pool responding to the salient item which preferentially drives the activity of the inhibitory pool earlier during the stimulation period. As a consequence, by the time enough recurrency is built up by another stimulated pool, the amount of inhibition that it receives is larger, thus reducing the likelihood of surpassing the inhibition currents flowing into the excitatory neurons. This is reflected in the fact that in the no saliency condition, the firing rates of most stimulated neuronal pools surpass that of the inhibitory neurons both simultaneously (or close in time) and earlier during the trial, whereas a graded access to such state of high firing rate is observed in the saliency condition. Importantly, under this paradigm, the described competition poses a limit to visual WM capacity since some neuronal pools are not able to reach the high firing rate state and, thus, cannot be held in WM, hence leading to a lower *e*WMC.

#### Time matters: the effect of varying the stimulation period

So far, the basic cooperative and competitive mechanisms giving rise to the storage of multiple items in visual WM have been identified. However, it has become evident that the dynamics of the different population activities during the stimulation period plays an important role in establishing an effective capacity limit to WM. It is therefore natural to address the issue of what is the effect of varying the stimulation period. To this end, different stimulation periods (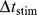) have been considered which include: 200 ms, 500 ms, and 1000 ms. Longer stimulation periods are not considered since rehearsal strategies might come into play, thus making it difficult to validate the model experimentally from human psychophysics. To isolate the effect of the stimulation period from that of saliency, the baseline condition in which all stimulated pools receive the same intensity is considered first and then compared to the condition with a visually salient item.

As can be seen from Fig. 6, as the stimulation time increases, the likelihood that those pools which have been stimulated reach and sustain the elevated firing rate required to be held in WM also increases. This fact is compatible with the existence of an upper boundary capacity limit governed by lateral inhibition during the delay period, as discussed by Edin et al. [27] but, interestingly, such limit can only be reached provided sufficient stimulation.

To this end, it is necessary to supply the stimulation for a sufficiently long period of time as previously reported in experimental works that make use of backward masking [42], [43]. We argue in next sections that, in fact, what matters is the joint effect of the stimulation intensity and stimulation period variables. However, from the results so far discussed, this effect stresses again the dependence of the *e*WMC on the dynamics of the system during the stimulation period, and not only on its behaviour at the stationary state.

It is worth noting that the role of visual saliency on WM function also varies as a function of time. Should the stimulation period be too short for allowing any stimulated selective neurons to reach a regime of high firing rate in the no saliency condition, the additional intensity received in the saliency condition by the neuronal pool selective to the salient item will facilitate better performances on trials in which the test item coincides with the salient one. This, however, would occur at the cost of not encoding the non-salient items. This is the case for the stimulation period 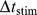 = 200 ms at the selected working point as can be seen from Fig. 6A. In terms of predicted performance, better overall performances in the saliency condition than in the no saliency condition would therefore be found. However, for longer stimulation periods, in the presence of visual saliency, the neuronal pool selective to the salient item would dominate the competition between the different stimulated pools, thus preventing the other stimulated selective pools from reaching the high firing rate state. In the absence of saliency, however, these other stimulated selective pools would have a higher chance of being correctly encoded into the WM system. From a statistical perspective, significant effects of saliency were encountered for all stimulation periods: 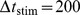 ms (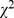
(2, 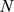 = 100) = 30.727, 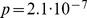), 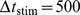 ms (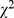
(2, 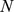 = 100) = 53.04, 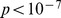), 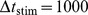 ms (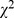
(2, 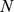 = 100) = 19.945, 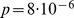). It is worth noting that the most conservative criterion when performing the 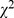 goodness of fit test has always been considered by setting the expected distribution to that one providing the smallest 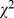 estimate.

#### Network size effects

We have also assessed the effect of varying the network size in the model because it is only through training that more neurons can be recruited to selectively respond to a particular stimulus or solve a given task. Thus, this is a parameter that may vary among subjects and therefore is prone to carry some variability in *e*WM capacity. Three network sizes have been considered in this study, namely 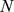 = 2500, 5000 and 10000 neurons. All simulations have been repeated for all of the previously studied stimulation periods (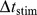 = 200 ms, 500 ms, and 1000 ms) in order to assess the concerted action of both variables.

**Figure 7:**
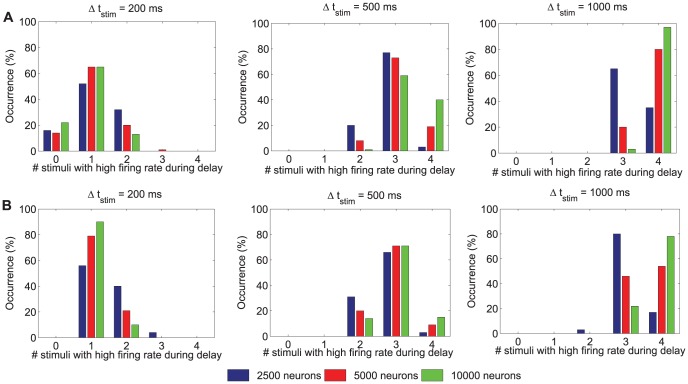
Distribution of number of items maintained in WM as a function of network size. Histograms illustrating the percentage of trials in which multiple items show high activity during the last 300 ms of the delay period for different stimulation periods: 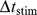 = 200 ms, 500 ms and 1000 ms and different network sizes 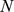 = 2500, 5000 and 10000 neurons. **A** Four pools receive an additional external stimulation 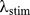 = 60 Hz, and **B** the pool selective to the salient item receives an external stimulation 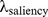 = 100 Hz while the remaining three stimulated pools receive only 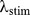 = 60 Hz. The network parameters employed in these simulations are those shown in Table 1. The results illustrate that *e*WMC increases when the stimulation period is increased for intermediate (500 ms) and long (1000 ms) stimulation periods, and is reduced in the presence of saliency. This tendency is stronger for larger network sizes as a consequence of the reduced finite size noise. For short stimulation periods (200 ms), the competition processes in the saliency condition favours a winner-take-all type of behaviour for the salient item.

**Figure 8:**
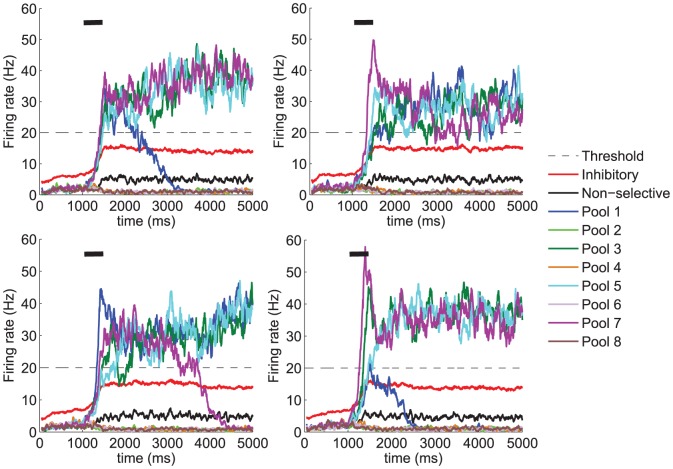
Firing rates predicted by the model for smaller network sizes. Results from the simulations of the full spiking model with 2500 neurons in four different trials in the baseline condition with no visual saliency and four pools (pool 1, pool 3, pool 5 and pool 7) receiving an external stimulation 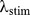 = 60 Hz. The results illustrate how smaller networks present larger finite size noise, which may lead to spontaneous memory losses throughout the delay period, and thus, reduce the *e*WMC of the system.

The same simulation parameters used in the previous studies, apart from the network size, have been considered in this study. The results obtained (see Fig. 7) indicate that for larger networks high *e*WM capacity states (e.g. 4 items) are more often reached than in the case of smaller networks for which lower *e*WM capacity states are more common. This is a consequence of finite size noise in the network which increases as network size decreases. Our results predict less stable high load states in the noisier conditions. This would imply that memories which have been correctly encoded and initially maintained in WM may be spontaneously lost throughout the delay period. This, in fact, is compatible with the notion that regardless of having reached the high firing rate state necessary to be maintained in memory (i.e. successful encoding), noise may provoke excursions from the basin of attraction of the higher load states into lower load attractors. The dynamical evolution of the neuronal firing rates shown in Fig. 8 (especially when compared to the results shown in Fig. 5A corresponding to a network size 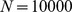) support such an interpretation. This is further confirmed from a statistical perspective since a 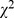 goodness of fit test (with Yates' correction) revealed significant differences 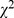
(2, 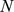 = 100) = 29.344, 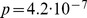) between the distributions corresponding to network sizes 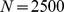 and 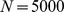 and also between the distributions corresponding to network sizes 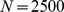 and 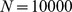 (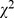
(1, 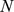 = 100) = 55.1, 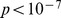) for 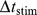 = 500 ms, which is the case illustrated in Figs. 8 and 5A.

These results further suggest that the limitation derived from having smaller networks can be compensated to some extent by increasing the stimulation period (or the stimulation amplitude as will be discussed later). This can be seen by comparing, for instance, the results obtained for a network size 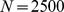 when 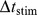 = 1000 ms, and 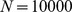 when 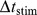 = 500 ms since no significant differences are revealed in this case (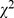
(1, 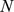 = 100) = 0.844, 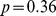). However, there seems to exist an upper boundary beyond which despite a successful encoding into the WM system, performance cannot be improved by acting only at the encoding level. Other strategies preventing memory loss should then be considered in order to compensate for the increased noise in the system.

The effect of saliency observed in this study provides further support to the conclusions from the previously presented results. For sufficiently long stimulation periods, salient items are preferentially maintained in WM during the delay period at the cost of not keeping as many non-salient items, thus reducing overall performance. A 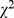 goodness of fit test (with Yates' correction) again reveals significant differences (in the most conservative case) between the saliency and no saliency condition for all network sizes (e.g. for 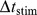 = 500 ms): 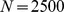 (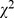
(1, 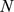 = 100) = 5.154, 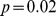), 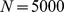 (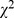
(2, 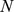 = 100) = 21.31, 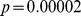), and 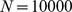 (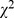
(2, 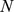 = 100) = 53.04, 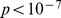).

For short stimulation periods (e.g. 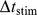 = 200 ms), the neuronal pool selective to the salient item clearly dominates the dynamics of the complete system and this tends to be the item preferentially maintained in WM throughout the delay period. This trend is more pronounced for larger networks in which the visually salient item very rapidly reaches the high firing rate state and stays in such state with small rate fluctuations.

#### Encoding memories into WM: the stimulation time/intensity tradeoff

**Figure 9:**
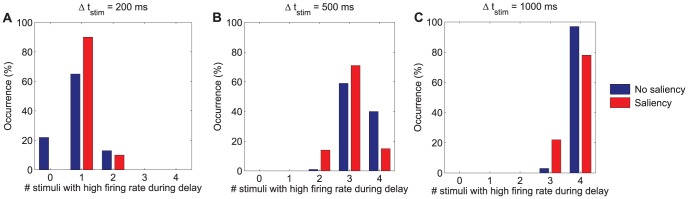
Distribution of number of items maintained in visual WM as a function of the additional external stimulation 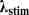
 **.** Histograms indicating the percentage of trials in which multiple items show high activity during the last 300 ms of the delay period for different stimulation periods: 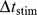 = 200 ms, 500 ms and 1000 ms, and different levels of additional external stimulation (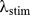 = 40, 60, and 80 Hz) received by the pools selective to the items in the visual display. **A** Four pools receive an external stimulation 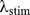 and **B** the pool selective to the salient item receives an external stimulation 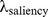 = 100 Hz while the remaining three stimulated pools receive only 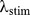. The network parameters employed in these simulations are those shown in Table 1. The results show that increasing the administered external stimulation 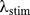 to all pools leads to an improvement in performace since the number of items held in visual WM increases overall. It is worth noting that the magnitude of the saliency effect is reduced for larger values of 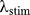 because the difference 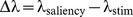 is smaller. The general trend observed in previous studies is, however, maintained in that the presence of a visually salient item in a display tends to reduce the number of items that are held in visual WM.

As previously suggested by Fig. 3, performance, and therefore the *e*WMC, may be enhanced by increasing the intensity of the external stimulation applied to the selective neurons. This effect is further analysed in Fig. 9 by varying the intensity that each pool receives and considering three different baseline intensities: 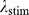 = 40, 60 and 80 Hz. It is generally difficult to establish a mapping between magnitudes such as visual stimulus contrast, response level relative to difficulty of encoding or total number of neurons encoding a particular item, and intensity of the external stimulation reaching the neuronal network. In this section, we will not attempt to establish such a mapping. We will, in contrast, consider such intensity simply as a variable in the model which may later be subject to interpretation. For the current study, and based on our previous results (see Fig. 3), we focus on intensity values for which behavioural differences exist. The neural mechanisms underlying the predicted behavioural differences will then be studied.

As can be seen from Fig. 9A, the proportion of trials in which all four stimulated items are correctly encoded and maintained in visual WM increases considerably by increasing the stimulation intensity 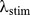. In fact, for a stimulation period of 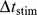 = 500 ms, the four stimulated items are held in WM for 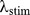 = 80 Hz, in contrast to the results found for the same stimulation period for an stimulation intensity 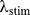 = 60 Hz. In contrast, for smaller intensities (e.g. 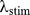 = 40 Hz), the proportion of trials reaching such state is smaller even for long stimulation periods. Since the differences emerging in this study between the different conditions are remarkable, no statistical analyses have been performed. From the neuronal point of view, these results can be interpreted by considering that the four stimulated pools compete to reach an elevated firing rate, which is, in fact, facilitated by the administered external stimulation. The larger the administered intensity, the more likely it will reach the attractor with an elevated firing rate. Thus, different intensities will leave the system in different basins of attraction corresponding to the different multistable states. However, upon retrieval of such stimulation, recurrency may not suffice to surpass the inhibition recruited throughout the stimulation period, which might in fact imply memory losses throughout the delay period. This is more likely to happen for larger intensities as can be seen from Fig. 3 when the case 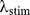 = 100 Hz is considered and compared to the results for 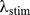 = 80 Hz.

Moreover, from these results it appears that increasing the stimulation intensity may have similar effects to increasing the stimulation period (i.e. increase *e*WMC). The administration of stronger stimulation facilitates a faster integration of activity whereas a more sustained but less strong stimulation can lead to similar results provided an smaller external stimulation is applied during a sufficiently long period. This leads us to hypothesise that is the concerted action of stimulation time and intensity which will determine the encoding of the items in WM. As can be also seen from these results (Fig. 9B), the impact of the upregulation associated with saliency is relative to the baseline condition, that is, the larger the difference between 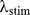 and 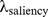, the larger will the difference in performance be. However, as will be discussed later, this effect does not only depend on the absolute difference but also on the relative difference between the two intensities, as is modulated by the baseline intensity level.

### Behavioural predictions and comparison with preliminary results

**Figure 10:**
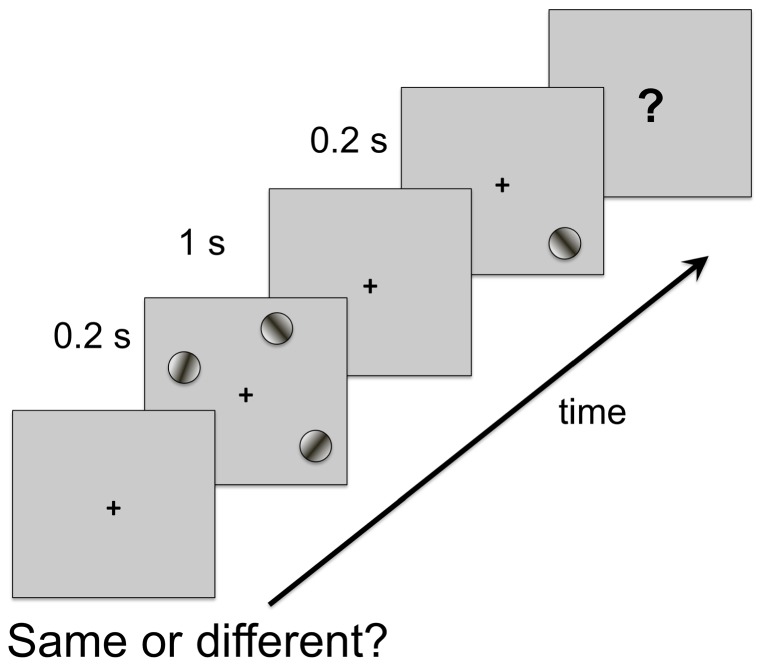
Experimental setup: task timeline. A stimulus set (“memory set”), consisting of 1 to 4 oriented Gabor stimuli, was shown for 200 ms in order to discourage subjects from making saccadic eye movements to scan the individual items. Trials were started by a button press and the first stimulus frame was only displayed after a variable delay time (500–700 ms). A fixation point was maintained at the center of the screen throughout each block of trials. The orientation of each Gabor stimulus in the memory set was one of eight possible orientations (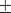
10, 20, 30 or 40 degrees from the vertical). A blank delay of 1000 ms followed the display of the memory set. Then, one probe stimulus (“test stimulus”) was shown for 200 ms. The test stimulus was identical to the Gabor patch at the same location in the memory set on “same trials”, whereas its orientation was mirror-reversed across the vertical on “different trials”. In the baseline condition, the Gabor stimuli had identical contrast and size (30% of full contrast). Separate blocks of trials were run in which the saliency of one item was manipulated by either increasing its bottom-up or top-down saliency. In the bottom-up saliency manipulation, the visual contrast with the background and/or the size of the Gabor stimulus was increased. Top-down saliency was manipulated by adding a memory-guided saccade task. To this end, a red dot was presented, along with the fixation point, at the beginning of the trials and participants were instructed to memorise this location in order to make a saccade there once the central fixation point was removed. Adapted with permission from Melcher and Piazza [19].

Finally, after having presented the proposed model and investigated its main features and predictions, we revisit the experiments by Melcher and Piazza [19] (which originally motivated the model) in order to assess whether our predictions and proposed neuronal mechanisms are compatible with the experimental observations. In these experiments, the stimuli used in the visual WM task were Gabor stimuli (oriented contrast gratings windowed by a Gaussian function) displayed against a mean grey background. Each Gabor stimulus subtended 1° in visual angle and was located in one of 16 positions in a 4
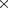
4 (8°
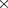
8°) grid centered at a fixation point displayed in black near the center of the screen. In the baseline condition, Gabor stimuli were shown at 30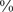 of full contrast (or up to 100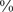 contrast in the high saliency condition) against a mean grey background. A sketch of the experimental set-up can be seen in Fig. 10.

**Figure 11:**
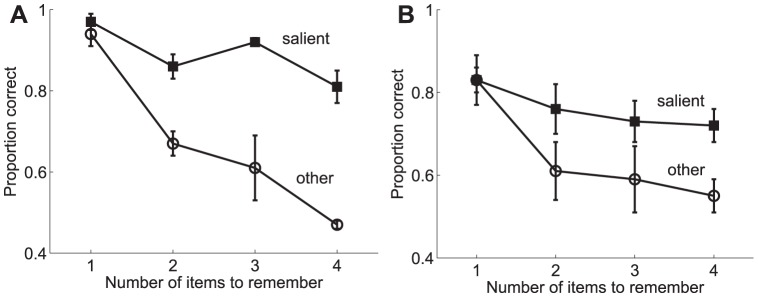
Influence of saliency on behavioural performace in a visual WM task. Proportion of correct responses for trials in which: **A** the bottom-up saliency of one of the items in the display was defined by manipulating the visual contrast, and **B** the top-down saliency of one of the items in the display resulted from an item appearing at a task-relevant location. Performance results for those trials in which the test stimulus is the salient item are distinguished from those in which the test stimulus is a non-salient item (denoted as “other” in the figure) to assess the influence of saliency on behavioural performance. Error bars show one standard error of the mean. Adapted with permission from Melcher and Piazza [19].

**Figure 12:**
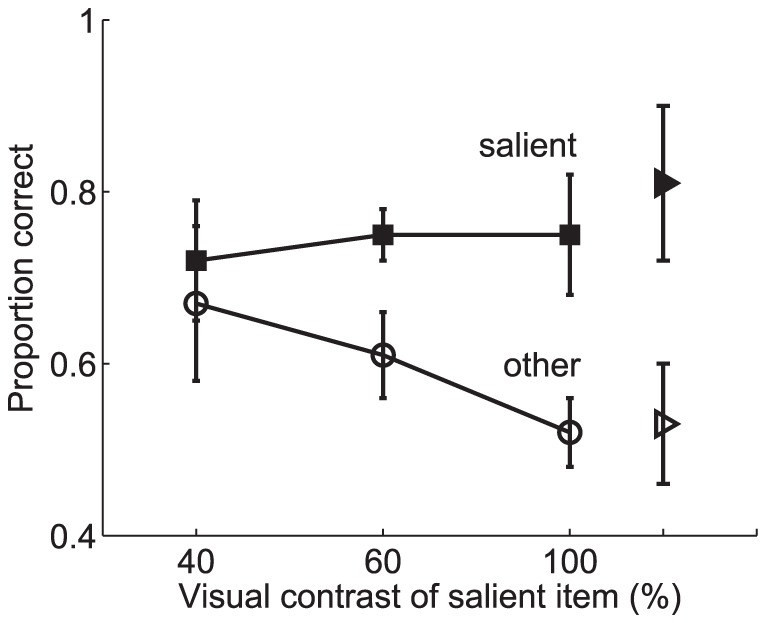
Influence of bottom-up saliency level on performance in a visual WM task. Proportion of correct responses for trials in which the bottom-up visual saliency of one item in the display was manipulated at different levels of visual contrast (also size in the case of the triangles). The number of items (set size) was held constant at three. Performance results for those trials in which the test stimulus is the salient item are distinguished from those in which the test stimulus is a non-salient item (denoted as “other” in the figure) to assess the influence of saliency level on behavioural performance. Error bars show one standard error of the mean. Adapted with permission from Melcher and Piazza [19].

As previously outlined, the results from these experiments (shown in Fig. 11 and Fig. 12) led to the following conclusions: 1) memory for the most salient item remained high, independent of set size, while performance for the non-salient item dropped with set size, and 2) both bottom-up and top-down saliency influenced visual WM in a similar way. They also studied the effect of the difference in contrast between the salient item and the remaining ones and showed that it increased as a function of the difference. It was concluded (see Fig. 12) that memory for the salient item remained relatively constant while performance for the non-salient items decreased as the relative saliency difference increased.

Before discussing the model predictions, two issues must nonetheless be stressed. On one hand, the proposed model is a model of object WM that specifically learns a set of objects. In contrast, Melcher and Piazza's experiment consider up to 4 stimuli which appear at different spatial locations and which belong to one of two categories (i.e. positive or negative orientation with regard to the vertical). Our object WM model has been adapted to these experiments by considering a set of populations which respond to the what/where conjunction. On the other hand, it is also worth noting that, in order to compare the model predictions with behavioural performances obtained from human subjects, a decision stage based on the neuronal activities of the pools during the delay period must be assumed. In particular, for the change detection paradigm considered by Melcher and Piazza [19], the decision module acts as follows: 1) it reads out the neuronal activity during the last part of the delay period (i.e. last 200 ms) and then 2) makes a decision based on the gathered evidence such that true positives (i.e. stable states with high sustained firing rate corresponding to stimulated pools) are always correctly identified whereas, in the absence of evidence, a random decision is made. Specifically, the proportion of correct (PC) answers is calculated based on the following premises:

- If the average response of the neural population selective to the test item is above a given threshold (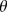 = 20 Hz, in this study), then, it is considered that the target is correctly held in memory and detection is error-free. It is worth noting that fake memories (i.e. maintenance of non-stimulated items) have not been observed in the regime in which the network operates, thus meaning that only previously seen items are, in fact, maintained in WM.
- If neither the neural population selective to the test item nor the neural population selective to the mirrored target exhibit a response above threshold, then it is considered that there is not enough evidence for any informed response and the subject will decide at random. Provided this is a change detection task, chance level is set at 50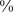.

For each condition (e.g. saliency level or memory set size), 100 simulations have been performed. These correspond to independent measures of the neuronal firing rate activity of the system (i.e. a set of ten neurons representative of the activity in each pool, since all neurons in the same pool become indistinguishable provided the selected connectivity structure) for each condition. In order to have an estimate of the proportion correct predicted by the model together with its variance, for each trial, the test part has been simulated also 100 times, such that, for each simulation derived from the full spiking model, the target sample is randomly retrieved from the pool of items in the memory set.

**Figure 13:**
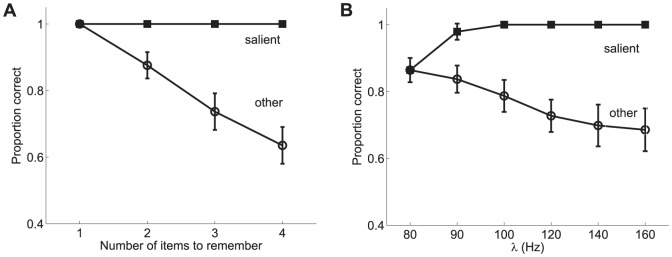
Model prediction of saliency influence on proportion correct in a visual WM task. **A** Performance predicted by the model in a change detection task akin to the experiments in [19] (also see Fig. 11) as a function of set size. The pool selective to the salient stimulus receives an external stimulation 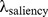 = 120 Hz whereas the pools that are not selective to the salient stimulus but are stimulated receive an external stimulation 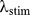 = 80 Hz during the 200 ms stimulation period. **B** Performance predicted by the model in a change detection task akin to the experiments in [19] (also see Fig. 12) as a function of the saliency level. In the model, visual saliency is introduced by means of an upregulation of the neuronal responses to the item. To this end, different levels of additional external stimulation are considered: 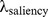 = 80, 90, 100, 120, 140 and 160 Hz for set size 3. In both cases, the results are assessed separately for the trials in which the target item corresponds to the salient item and those which do not coincide with the salient item (i.e. other). The network parameters employed in these simulations are those shown in Table 1. The results suggest a good qualitative conformance with the experimental results shown in Fig. 11 and Fig. 12. It is worth noting that no specific tuning of the network parameters has been sought to reproduce such results. The same parameters used in the previous studies, which reproduce the main results from the available literature, have also been used in this case.

As previously stated, Melcher and Piazza showed in [19] that visual WM capacity was influenced by changes in the relative saliency of the items. Memory for the most salient item remained high, independent of increased set size, while performance for the non-salient item dropped precipitously with set size (Fig. 11A). A similar trend was found in trials in which one item was more salient because it was presented at the saccade target location (Fig. 11B). Thus, both bottom-up and top-down saliency influenced visual WM in similar ways. The results obtained from the proposed model are shown in Fig. 13A. It is worth noting that from the standpoint of the model there is no difference between bottom-up and top-down saliency since both imply an upregulation of the firing rate. As can be observed, a good qualitative fit is obtained between the behavioural data and the predictions of the proposed model. No effort has been done in tuning the model parameters in order to obtain a good quantitative fit but rather the same parameters used in the previous studies have been considered here. However, remarkably, the results reproduce the main trends found in the experiments.

These results are, of course, in agreement with the hypothesis that WM resources are limited and shared (although not necessarily equally distributed) among the different items in the visual field. Thus, should one of them recruit further resources that will imply a poorer performance for the remaining items. Melcher and Piazza [19] also studied the effect of the difference in contrast between the salient item and the remaining ones. They showed that such effect increased as a function of the difference, with the largest effect found when both the contrast and the size of the salient item were increased (Fig. 12, triangles). In terms of the proportion of correct responses (Fig. 13B), memory for the salient item remained relatively constant after an initial increase for smaller set sizes while performance for the non-salient items decreased as saliency increased.

**Figure 14:**
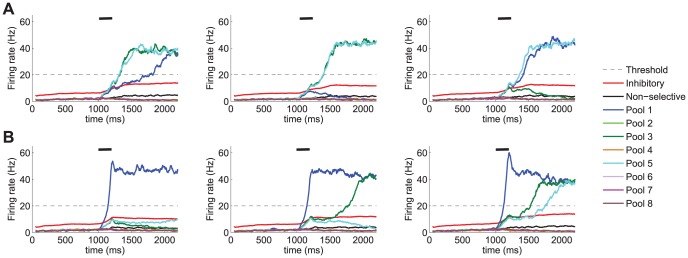
Model predictions of firing rates. Illustration of three possible neuronal behaviours observed when 3 pools are simultaneously stimulated. The stimulation period is represented by means of a thick back line. Noise introduces a stochastic component which is translated into a distribution of possible neuronal behaviours. **A** Trials in which no saliency effects are present and the three pools selective to the visual items in the display (pool 1, pool 3 and pool 5) receive an intensity 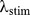 = 80 Hz, and **B** trials in which one of the three items in the visual display is salient and the pool selective to such item (pool 1) receives an external current 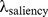 = 120 Hz instead of 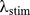 = 80 Hz. The response of the pool selective to the salient stimulus always reaches an elevated firing rate which, in turn, recruits inhibition and may prevent other pools from reaching such elevated firing rates, and thus, reduces the *e*WMC.

The neural substrate of such claim is analysed in Fig. 14, showing the firing rates of all the neural pools involved in the simulations in the case when three pools are simultaneously stimulated with equal intensity (Fig. 14A) or with one of them receiving a higher stimulation (Fig. 14B). As can be seen, in the salient condition (Fig. 14B), the competition is led by pool 1, responsive to the salient item, and thus receiving the highest excitation (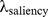 = 120 Hz). Overall performance drops in the saliency condition since pool 1 dominates the activity of the system and prevents, more often than in the no saliency condition, other pools from reaching an elevated firing rate. Again, in the no saliency condition, the interplay of cooperative and competitive processes during the stimulation period imply that several pools will be able to reach the required sustained firing rate more often and also almost simultaneously, whereas a graded encoding is seen in the saliency condition.

**Figure 15:**
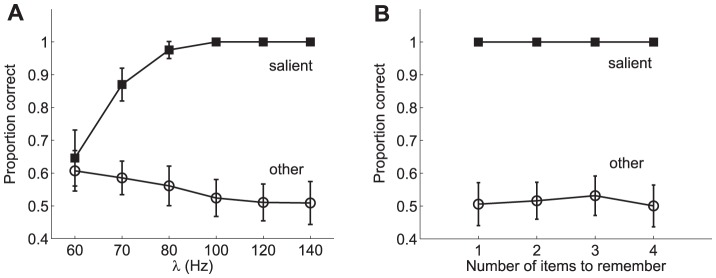
Performance dependence on stimulation amplitude at baseline level. The stimulation amplitude at baseline level 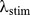 is a limiting factor of *e*WMC. Not only a sufficiently large 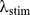 value is necessary to reach the full WM capacity of the network but also the absolute difference between 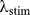 and 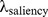 has different effects depending on the baseline level 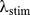. **A** Performance predicted by the model in a change detection task akin to the experiments in [19] as a function of the saliency level. In the model, visual saliency is introduced by means of an upregulation of the neuronal responses to the item. To this end, different levels of additional external stimulation are considered: 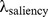 = 60, 70, 80, 100, 120 and 140 Hz for set size 3. **B** Performance predicted by the model in a change detection task akin to the experiments in [19] as a function of set size. The pool selective to the salient stimulus receives an external stimulation 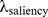 = 100 Hz whereas the pools that are not selective to the salient stimulus but are stimulated receive an external stimulation 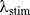 = 60 Hz during the 200 ms stimulation period. This corresponds, in fact, to a particular working point of the graph in **A** for a varying set size. In this case, the salient item is always maintained in working memory throughout the delay period but random performance is obtained for non-salient items, thus suggesting a winner salient item in a winner-take-all network. In both plots, the results are assessed separately for the trials in which the target item corresponds to the salient item and those which do not coincide with the salient item (i.e. other). The network parameters employed in these simulations are those shown in Table 1.

The effect of saliency on performance varies with the difference arising from the external stimulation 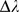 (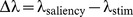), and also, as can be seen from Fig. 15 is relative to the baseline stimulation for non-salient items. Thus, should one attempt to tune the values 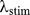 and 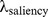 to closely match the experimental results, it is worth noting that their relative difference should also be considered. This can be interpreted in the following way. Different tasks might demonstrate different encoding difficulties (e.g. colour discrimination vs shape discrimination). Thus, the amount of external stimulation (characterised either by the intensity or the integration of evidence during the stimulation period) may vary performance. Increasing the baseline stimulation to all pools, makes them more competitive (and then more able) to reach the required high activation state.

Similarly, the opposite is also true: taking away stimulation enables only the pool selective to the salient item to reach such a state. The specific value for which this happens depends on the balance between inhibition and excitation that takes the network away from the almost balanced condition into an inhibition dominant regime. This is a parameter that can be adjusted for each specific task at hand.

Therefore, similar absolute differences in external stimulation do result in different performances. As can be observed from Fig. 15, there may even exist a condition relating 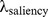 and 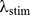 for a particular value of 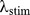 from which the salient item completely dominates the competition and the network behaves as a classical winner-take all network. As a consequence, performance is driven in that case by the salient stimulus with 100% correct trials for the visually salient item and chance level performance for the remaining non-salient items, as illustrated in Fig. 15B. This could be interpreted considering that attention is locked to the salient item, upregulating the response to such item while filtering out the ignored items. This is in agreement with the experimental results shown in Everling et al. [41]. Thus, the extent to which saliency locks visual attention to a particular item seems to determine the performance in the different conditions.

## Discussion

This study reports the results from a computational investigation into the neuronal mechanisms underlying the visual WM capacity limits observed in human psychophysics while attempting to provide new evidence that sheds some light onto the different views defended by the two main competing psychological theories dealing with visual WM capacity: the fixed capacity (“slot”) models (e.g. [9]), and the dynamical allocation of resources models (e.g. [5]). To this end, an experimental paradigm that exploits the potential of visual saliency to facilitate a redistribution of resources, proposed by Melcher and Piazza [19], has been considered.

Numerous studies suggest that visual saliency helps to explain the allocation of visual attention to stimuli in a scene. However, since the task consists of remembering as many objects as possible, such visual attention would be distributed among several objects and would only be modulated by the relative saliency of the salient item compared to the non-salient items. Visual saliency has therefore been characterised in the computational model by means of an upregulation of the firing response patterns of selective neurons, a notion which is compatible with available neurophysiological evidence [41].

The predictions of our model lie somewhere on a continuum between pure slot and pure shared resources models. In agreement with the discussion presented in [5], our results suggest that visual saliency acts as a “gatekeeper” determining which visual information is given priority for storage in WM. The results presented in this work are thus compatible with the existence of dynamic shifts of limited WM resources proposed by Bays and Husain [5]. Furthermore, our model implies that this might occur by biasing competitive interactions in cortical regions which would thus shape visual WM function. As already discussed, in our study the dynamic allocation of resources would be accomplished by rendering a visual stimulus salient. However, from the standpoint of the model, it is irrelevant what is the origin of such saliency, which may in fact stem from either endogenous or exogenous sources as well as from any concerted action of both. Furthermore, the experimental observation and model prediction of the bottom-up saliency effect on WM capacity would not have been directly predicted by either capacity or resource models, but instead fits in well with theories suggesting that limited capacity results from competition between items [44]. A similar problem was addressed by Bays and Husain [5] by assessing the effect of saccadic eye movements on memory. The results therein obtained are compatible with the explanation that our model proposes since visual attention precedes saccadic eye movements and an upregulation of the neuronal response to the salient item would also occur in such scenario.

In contrast to the predictions of fixed capacity (“slot”) models [9], we found that the number of items that could be remembered was not fixed, but varied with the relative saliency of the items. Inconsistent with the resource model [5], however, we found that an upper boundary capacity limit exists as predicted by the fixed capacity model. This upper boundary would only depend on the network properties. Nonetheless, it could only be achieved under specific conditions, governed by the experimental protocol, which determines the dynamics of the neuronal system during the encoding stage into WM.

A key question that clearly emerges from this computational study is the following: what do we actually mean when we refer to WM capacity? Can we talk about different WM capacities depending on the experiment at hand, or even, the particular experimental protocol (e.g. stimulation time, saliency level, etc)? Or perhaps the use of the term “capacity” refers to a maximal upper limit under optimal conditions rather than typical performance in any particular task? Following the results from our study, we suggest that WM capacity limit should be used to refer to the absolute upper boundary magnitude, presumably hard wired by the network itself, that establishes the maximum number of items that can be possibly held in WM. This would correspond in the attractor picture to the upper boundary value predicted by Edin et al. [27]. In contrast, we propose the term *e*WMC, contingent upon the experimental conditions (e.g. nature of the stimuli, stimulation period, etc) and largely dependent on the stimulation protocol and its accompanying neuronal dynamics, to refer to the WM capacity observed in each experiment. This idea would be a natural consequence of the models compatible with the dynamic allocation of resources but would differ from the proposed model in Bays and Husain [5] in the existence of the upper boundary capacity. In terms of the attractor picture, this implies that the dynamics of the system during the transient period, largely ignored by previous studies, will be responsible for which basin of attraction the system enters, and thus, how many objects can be actually held in WM. This allows us to identify two relevant stages in WM function in delayed match-to-sample experiments: the encoding stage (associated with the stimulation period) and the maintenance stage (associated with the delay period). In fact, the encoding stage is of outmost importance in determining the differences between the WM capacity limit and the *e*WMC.

While a few studies of visual WM have examined the role of stimulus factors in determining capacity, this mainly involved changing the complexity or nature of all the items simultaneously [9]. Furthermore, it is often the case that when different types of stimuli are considered, the stimulation period is also changed in the experimental design. We found, however, that making one item more salient had an enormous impact on the neural dynamics and may lead to a reduction of the overall capacity supporting the idea established by Bays and Husain [5] that WM resources can be shifted flexibly between objects, with allocation biased by selective attention.

Interestingly, some experimental studies suggest that changing the presentation time does not limit WM capacity [8]. Nonetheless, although it is often assumed that the effective duration of the stimulus is not important for visual WM tasks, Gegenfurtner and Sperling [42] showed that visual masking influenced performance in a verbal WM task. In particular, reducing the inter-stimulus interval between the targets (letters) and the mask reduced performance. They estimated that each item took around 30 ms to be consolidated into WM. This idea was followed up in a recent study of visual WM [43] which reported a similar trend with coloured squares as stimuli and backward masking. Together both studies suggest that reaching full visual WM capacity requires at least 150–200 ms.

Similarly, it has been reported that reducing the visibility of stimuli [45] or increasing the complexity of the stimulus [6] both reduce visual WM. In the case of visibility, it has been shown that lowering contrast reduces performance in enumerating the number of items in the display. Although this was not studied with a change detection task, it has been suggested that enumeration and visual WM share similar processes of object individuation and show a similar capacity limit of around 3–4 items [46]. The report that increasing stimulus complexity reduced visual WM capacity is also consistent with our model, in that more complex items would tend to activate a smaller number of neurons which preferentially respond to that particular combination of features. A coloured square, for example, would tend to activate more feature-specific neurons as a preferred stimulus than would a complex artificially-generated shape. We further speculate that the intensity 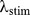 used in our model may in fact intrinsically reflect the nature (and complexity) of the items. Thus, simple attributes such as colour, overly represented in the visual cortex would have an associated large value 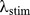 whereas more complex attributes such as shapes would have smaller associated intensities 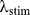 because less neurons would specifically respond to them. Should we assume such hypothesis, our model would predict that shorter stimulation periods would be required to reach the WM capacity limit for colours than shapes, something that is implicit in some experimental designs [9].

Our model also predicts that stimulus duration and stimulus strength interact in defining *e*WMC. Specifically, should the stimulation time be sufficient to set the system in a state corresponding to the highest possible load given by the WM capacity limit, having longer stimulation periods would not noticeably modify behavioural performance. However, should this stimulation period not suffice to such effects, then behavioural performance would decrease, leading to the conclusion that the *e*WMC decreases.

From the standpoint of our model, *e*WMC would be bound by a maximal WM capacity (akin to the limit proposed by fixed capacity models). This maximal WM capacity would be dependent only on the network properties, and thus, would be susceptible to the natural variations between different subjects. However, *e*WMC would also depend on aspects specific to the experimental design such as stimulation period or type of stimulus, and thus, in that sense would be contingent upon the task at hand. This interpretation might even lead us into the world of neurological diseases and, for instance, from a perspective of neurodegenerative diseases such as Alzheimer's disease (which implies neural mass loss), our model would allow us to speculate that lower WM capacities would be encountered for patients, as expected from our study with variable network sizes.

An aspect which is worth noting regarding the computational model is that no attempt has been made to include mechanisms that are able to account for the accuracy with which items are encoded and stored in WM. Previous computational studies have suggested that accuracy can be accounted for by considering continuous attractor models [47]. However, we presume that the emergence of bumps of activity would follow similar competitive mechanisms to those addressed in this study, and therefore, similar qualitative results are expected with visual saliency modulating the competition between these bumps instead of the competition between peaks of activity considered in our study.

By selecting a discrete object WM network, we admittedly address an oversimplified system. Nonetheless, such a system could be considered as a minimal model that preserves key aspects of WM function, thus allowing us to dive into the essential neuronal mechanisms that still reveal interesting insights. Systematic studies have been conducted to understand the effect of a number of variables, and their relation to experimentally manipulable parameters has been sought. By dealing with an object WM system, spatial information has not been taken into consideration. However, it is in general difficult to completely separate the processes in the ventral and dorsal visual pathways since any manipulation on the visual display will affect both dimensions (i.e. the spatial and feature/object dimensions). Future studies that address the interaction of both pathways are required to better understand WM function.

Furthermore, we also face the issue of how perceptual decisions are made, which is a common issue facing any study comparing a computational model of neural firing with behavioural data since no direct measures matching our simulation protocols are available yet. To provide an alternative indirect validation of the proposed model, in this study, we have adopted a naïve decision making model together with a simplified experimental protocol which allows theoretical predictions for preliminary comparisons with the data available in the literature. This decision stage is built on top of the network model as reads out the neuronal activity during the delay period. Nevertheless, only neurophysiological measures seeking the neural substrate of WM capacity limits will provide direct validations of computational models such as ours.

Fortunately, studies of monkey neurophysiology are starting to provide some evidence regarding storage of multiple items. In one such study, Warden and Miller [26] conclude that multiple objects are not stored in separate populations of PFC neurons but rather they are represented by a single population whose activity depends on the identity of the various objects and also encapsulates the temporal order of objects by varying the response strength of the neurons over time. This is in contrast with the much more persistent firing rates found by Amit et al. [38] following sequential stimulation of multiple items. However, these experiments contain a dynamic component which the proposed model does not address, i.e. the sequential stimulation, and therefore it is difficult to compare the two approaches.

A recent study by Buschmann et al. [28] specifically addresses the neural substrate of WM capacity limits. Interestingly, in agreement with our results, the authors discuss that elements from both discrete-resource models (i.e. the existence of an upper boundary limit) and flexible-resource models (i.e. gradual division of neuronal information among objects in the visual display) are at play in determining WM capacity. Furthermore, the importance of sensory encoding in determining capacity limits is also stressed in comparison with memory failure, in contrast to previous computational studies (e.g. [27]) which were mainly focused on item maintenance throughout the delay period as the only key aspect for establishing WM capacity limits. However, the authors hypothesise that there are two different networks each one in charge of encoding and storing the information from a hemifield. This differs from our model since we assume a single network with a WM capacity limit derived from the complete visual field. Although the authors provide suggestive evidence for such dual model, we would argue that the proposed experiment follows an experimental protocol that combines in a complex way visual information from the dorsal and ventral visual pathways. That is, monkeys are not only required to recognise whether a change had occurred but also they should both know where it had occurred and make a single directed saccade to the changed object. Thus, it could also well be the case that lateral inhibition between items located close to each other in space would be responsible from such apparent division in hemifields. This could be accounted for by a single network with a topographical and structured connectivity organisation sensitive to such spatial effects. Furthermore, as the authors discuss, the capacity bottleneck suggesting an hemifield separation is more grounded in PPC, where neuronal receptive fields are more restricted to a visual hemifield than in PFC. However, when only object WM is assessed, the role of PPC is utterly reduced. Finally, we argue that our model could, however, be adapted to reproduce their experimental results by setting the working point of the network in a regime in which the WM capacity limit is established to be two items since all other basic phenomena were already encapsulated by our proposed model.

Taken together, we introduce the notion of *e*WMC as a construct which emerges naturally from basic principles of the proposed model and provides both a general framework to investigate WM function and, importantly, a plausible explanation of the mechanisms yielding WM capacity limits based on neural mechanisms. Finally, the model also makes specific firing rates and behavioural predictions and suggests how these can be related with manipulable experimental variables. In this sense, the study confirms, once again, the general accordance of attractor networks with neural processes but also offers predictions which may be used to guide, in a principled way, the design of experiments in order to further explore WM function.

## Methods

### Computational model description

The behaviour of the neurons is modelled by means of the leaky integrate-and-fire (LIF) model, in which the membrane potential 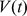 obeys the following differential equation:
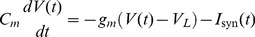
(1)where 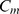 is the total membrane capacitance, 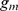 is the passive conductance, 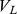 is the resting potential, and 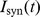 is the synaptic current that charges the neuron. In this work, four families of synapses have been considered. The recurrent excitatory postsynaptic currents (EPSCs) have two components, which are mediated by AMPA and NMDA receptors. In contrast, only AMPA receptors mediate external EPSCs and GABA receptors mediate the inhibitory components. As a result, the total synaptic current is defined as follows:
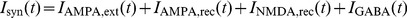
(2)where
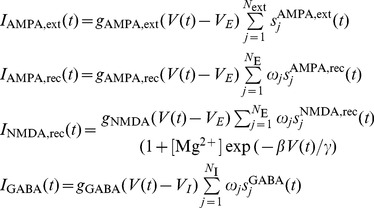
(3)


In these equations, 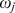 are dimensionless weights representing structured excitatory recurrent connections, and 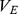 and 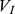 are the excitatory and inhibitory reversal potentials, respectively. The NMDA current is potential dependent and is controlled by the extracellular concentration of magnesium 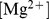. The dynamics of the gating variables 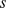 obey the following differential equations:
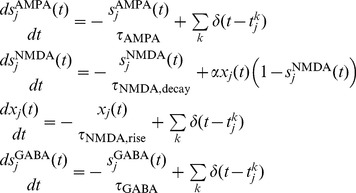
(4)where the rise time of AMPA and GABA currents is neglected, and recurrent excitation is assumed to be largely mediated by NMDA receptors, thus facilitating more stable mnemonic activity. The different parameters used in the simulation studies are shown in Table 1.

### Connectivity structure

The network consists of 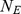 (80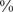) excitatory pyramidal neurons, and 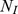 (20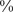) inhibitory interneurons and is all-to-all connected. The coupling strengths are not varied independently but an homeostatic mechanism is invoked since the coexistence of potentiation and depression is a regulatory mechanism necessary to keep the network activity stable. Such homeostatic mechanism, which relates 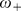 and 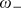, requires that the average excitatory-to-excitatory synaptic strength remains approximately constant during learning. In contrast to the original homeostatic condition considered in Brunel and Wang [30]:
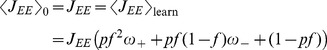
(5)where 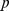 denotes the number of selective excitatory subpopulations (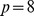 throughout this study), 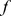 is the coding level or fraction of neurons in each excitatory subpopulation, and 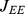 is the baseline synaptic strength between excitatory neurons prior to learing, a small value 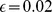 has been added to the definition of 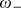, thereby enhancing the general excitability of the network without destabilising the baseline spontaneous state. Thus, 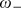 is determined by 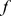, 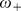, and 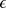 through the following equation:
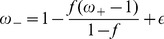
(6)


Since the model considers a fully connected network, each neuron receives 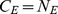 excitatory contacts from pyramidal cells and 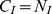 inhibitory contacts from interneurons. Furthermore, all the neurons in the network also receive 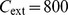 excitatory connections from outside the network. Such external activity arrives at each synapsis with a rate of 3 Hz [30], thus leading to an overall external rate 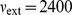 Hz. This is in agreement with the regime in which the neocortex is found to operate [48] since, as investigated by Stetter [49], the network responds to small perturbations 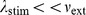 that ride on top of the large background signal by strongly and selectively amplifying them.

### Simulations

The spiking simulations conducted in this study consisted of 100 trials for each condition. The simulation protocol did not consider the display of the test item and, thus, the simulations ended prior to its presentation. Simplified decision making models were subsequently used to obtain performance estimates. A second-order Runge-Kutta routine, with a time step of 0.02 ms was used to numerically integrate the coupled differential equations describing the dynamics of the system. In order to calculate the firing rates, all spikes corresponding to each neural population were counted over a sliding window of width 50 ms, which was shifted with time a step of 5 ms. The average population firing rate was then obtained by dividing the total number of spikes by the number of neurons in the corresponding population and the window width. Numerical integration of the mean field equations was performed using an Euler routine with a step size of 0.1 ms.

### Model-based performance estimates

Two model-based performance estimates were used to assess behavioural performance from the neuronal activity of the selective pools during the delay period. The estimates differed on the protocol used to select the test item. Two possible scenarios were considered: 1) the test item was selected from the set of all 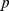 objects learned by the network (in this case the test item may or may not be present in the memory set) or 2) the test item is selected from the memory set. As previously stated, in the first scenario, correct responses may correspond to either the correct maintenance of an object in the memory set or the correct recall that an item was not present in the memory set. The proportion of correct responses in this scenario, 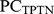, is defined in Eq.7.

In Eqs. 7 and 8, 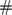
stim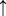 corresponds to the number of pools showing a persistently high firing rate after stimulating the WM system with a number of visual stimuli 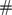
stim equal to the memory set size. The total number of objects learned by the network (i.e. 8 throughout this study) is denoted as 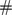
total stim. Since the system does not show any false memories in our case, the rate 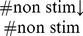 of pools with low firing rate (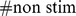) among those pools not stimulated (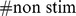) is always one.
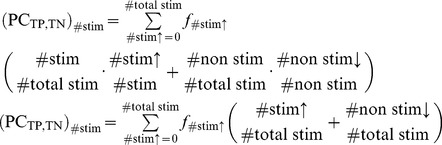
(7)


In the second scenario, correct responses would only correspond to the correct maintenance of an object in the memory set. The performance estimate in this case is 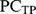 and is defined in Eq.8.
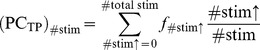
(8)


In both cases, 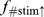 corresponds to the proportion of trials in which a given number of items 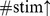 is maintained in WM for a particular memory set size.

## Supporting Information

Text S1 **Supplementary Methods.** Mean field approximation (following Brunel and Wang (2001)).
(PDF)
