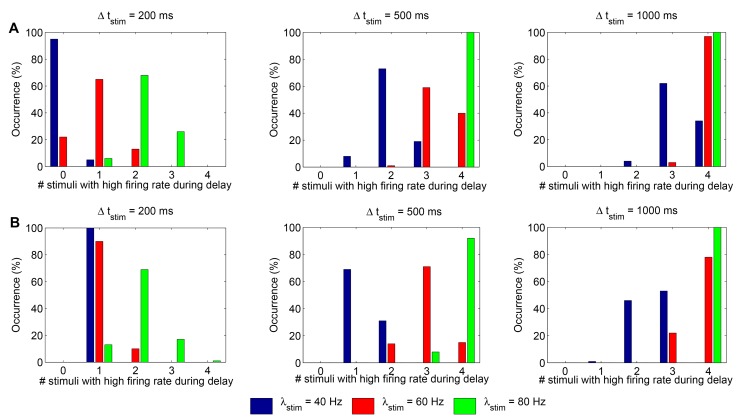# Correction: Effective Visual Working Memory Capacity: An Emergent Effect from the Neural Dynamics in an Attractor Network

**DOI:** 10.1371/annotation/85fd1653-3473-4218-89e2-9a0c60ea704e

**Published:** 2012-10-31

**Authors:** Laura Dempere-Marco, David P. Melcher, Gustavo Deco

Figure 9 is incorrect. The correct figure can be found here: 

**Figure:**