# Supplementary material for: Effective Visual Working Memory Capacity: An Emergent Effect from the Neural Dynamics in an Attractor Network
Source: PLoS One. 2012 Aug 29;7(8):e42719. doi: 10.1371/journal.pone.0042719 (PMC3430714; doi:10.1371/journal.pone.0042719)
Supplement: Text S1 — Supplementary Methods. Mean field approximation (following Brunel and Wang (2001)). (PDF) [file pone.0042719.s001.pdf]

Supplementary Methods

Mean field approximation

The mean field approximation provides a simplification of the integrate-and-fire dynamics considered in the spiking simulations, which are computationally expensive and are therefore not suitable for exhaustive parameter explorations. The details of the complete mean field analysis of neural networks with conductance-based synaptic inputs can be found in Brunel and Wang [1]. In this work, we have used the mean field approximation therein derived to scan the parameter space $\{\omega_I, \omega_+\}$. Following this approximation, the stationary dynamics of each neural population is described by the population transduction function. This provides the average population rates after a period of dynamical transients as a function of the average input current. Such stationary rates can be found as the stable solutions of a self-consistency condition and correspond to the stable attractors of the structured network.

In this formulation the potential of a neuron is calculated as:

$$\tau_x \frac{dV(t)}{dt} = -V(t) + \mu_x + \sigma_x \sqrt{\tau_x}\eta(t) \tag{S.1}$$

where $V(t)$ is the membrane potential, x labels the populations (i.e. activated selective subpopulations, non-activated selective subpopulations, nonselective cells and inhibitory cells), τ_x is the effective membrane time constant, μ_x is the mean value the membrane potential would have in the absence of spiking and fluctuations, σ_x measures the magnitude of the fluctuations and η is a Gaussian process with absolute exponentially decaying correlation function and time constant τ_{AMPA}. The magnitudes μ_x and σ_x are given by the following equations:

$$\mu_x = \frac{(T_{\text{ext}}\nu_{\text{ext}} + T_{\text{AMPA}} n_x^{\text{AMPA}} + \rho_1 n_x^{\text{NMDA}})\, V_{\text{E}} + \rho_2 n_x^{\text{NMDA}} \langle V \rangle + T_{\text{I}} n_x^{\text{GABA}} V_{\text{I}} + V_{\text{L}}}{S_x} \tag{S.2}$$

$$\sigma_x^2 = \frac{g_{\text{AMPA,ext}}^2 \left(\langle V \rangle - V_{\text{E}}\right)^2 N_{\text{ext}} \nu_{\text{ext}} \tau_{\text{AMPA}}^2 \tau_x}{g_m^2 \tau_m^2} \tag{S.3}$$

In these equations ν_{ext} corresponds to the external incoming spiking rate, ν_I is the spiking rate of the inhibitory population, and $\tau_m = C_m/g_m$ (whose value depends on whether excitatory or inhibitory cells are considered, see Table 1 in main text). The rest of variables are given by:

$$S_x = 1 + T_{\text{ext}}\nu_{\text{ext}} + T_{\text{AMPA}} n_x^{\text{AMPA}} + (\rho_1 + \rho_2) n_x^{\text{NMDA}} + T_I n_x^{\text{GABA}} \tag{S.4}$$

$$\tau_x = \frac{C_m}{g_m S_x} \tag{S.5}$$

$$T_{\text{ext}} = \frac{g_{\text{AMPA,ext}} \tau_{\text{AMPA}}}{g_m} \tag{S.6}$$

$$T_{\text{AMPA}} = \frac{g_{\text{AMPA,rec}} N_{\text{E}} \tau_{\text{AMPA}}}{g_m} \tag{S.7}$$

$$T_{\text{I}} = \frac{g_{\text{GABA}} N_{\text{I}} \tau_{\text{GABA}}}{g_m} \tag{S.8}$$

$$n_x^{\text{AMPA}} = \sum_{j=1}^{p} f_j \omega_{jx}^{\text{AMPA}} \nu_j \tag{S.9}$$

$$n_x^{\mathrm{GABA}} = \sum_{j=1}^{p} f_j \omega_{jx}^{\mathrm{GABA}} \nu_j \tag{S.10}$$

$$n_x^{\mathrm{NMDA}} = \sum_{j=1}^{p} f_j \omega_{jx}^{\mathrm{NMDA}} \Psi(\nu_j) \tag{S.11}$$

$$\Psi(\nu) = \frac{\nu \tau_{\mathrm{NMDA}}}{1 + \nu \tau_{\mathrm{NMDA}}} \left(1 + \frac{1}{1 + \nu \tau_{\mathrm{NMDA}}} \sum_{n=1}^{\infty} \frac{(-\alpha \tau_{\mathrm{NMDA,rise}})^n T_n(\nu)}{(n+1)!} \right) \tag{S.12}$$

$$T_n(\nu) = \sum_{k=0}^{n} (-1)^k \binom{n}{k} \frac{\tau_{\mathrm{NMDA,rise}}(1 + \nu \tau_{\mathrm{NMDA}})}{\tau_{\mathrm{NMDA,rise}}(1 + \nu \tau_{\mathrm{NMDA}}) + k \tau_{\mathrm{NMDA,decay}}} \tag{S.13}$$

$$\tau_{\mathrm{NMDA}} = \alpha \tau_{\mathrm{NMDA,rise}} \tau_{\mathrm{NMDA,decay}} \tag{S.14}$$

$$\rho_1 = \frac{g_{\mathrm{NMDA}} N_{\mathrm{E}}}{g_m J} \tag{S.15}$$

$$\rho_2 = \beta \frac{g_{\mathrm{NMDA}} N_{\mathrm{E}} (\langle V_x \rangle - V_{\mathrm{E}})(J-1)}{g_m J^2} \tag{S.16}$$

$$J = 1 + \gamma \exp(-\beta \langle V_x \rangle) \tag{S.17}$$

$$\langle V_x \rangle = \mu_x - (V_{\mathrm{thr}} - V_{\mathrm{reset}}) \nu_x \tau_x \tag{S.18}$$

where p denotes the number of selective excitatory subpopulations (i.e. eight throughout this study), f_x is the coding level or fraction of neurons in each excitatory subpopulation x, ω_{jx} is the synaptic weight of the connections from population x to population j, ν_x is the spiking rate of the excitatory subpopulation x, $\gamma = [\mathrm{Mg}^{2+}]/3.57$, $\beta = 0.062$, and the average membrane potential $\langle V_x \rangle$ has a value in the range $[-55, -50]$ mV. In this study, the synaptic weights described in Fig. 2 have been used. In particular, in those cases when $\omega_{jx}^{\mathrm{GABA}}$ is different from zero it always takes the value ω_I. Furthermore, $\omega_{jx}^{\mathrm{AMPA}} = \omega_{jx}^{\mathrm{NMDA}}$, which may take the values ω_+, ω_- or $\omega = 1$, depending on the neural populations x and j which are connected.

The average firing rates of the different populations in the network can be calculated in the mean field approximation from the solution of a set of n nonlinear equations which depend on μ_x and σ_x as follows:

$$\nu_x = \Phi(\mu_x, \sigma_x) \qquad x = 1, ..., n \tag{S.19}$$

where Φ is the transduction function of population x (i.e. the f-I curve), which enables the calculation of the output rate of population x in terms of its inputs, which in turn also depend on the spiking rates of all the rest of the populations. The transduction function is given by:

$$\Phi(\mu_x, \sigma_x) = \left(\tau_{rp} + \tau_x \int_{\beta(\mu_x, \sigma_x)}^{\alpha(\mu_x, \sigma_x)} du \sqrt{\pi} \exp(\mu^2)[1 + \mathrm{erf}(u)] \right)^{-1} \tag{S.20}$$

$$\alpha(\mu_x, \sigma_x) = \left(\frac{V_{\mathrm{thr}} - \mu_x}{\sigma_x} \right) \left(1 + 0.5 \frac{\tau_{\mathrm{AMPA}}}{\tau_x} \right) + 1.03 \sqrt{\frac{\tau_{\mathrm{AMPA}}}{\tau_x}} - 0.5 \frac{\tau_{\mathrm{AMPA}}}{\tau_x} \tag{S.21}$$

$$\beta(\mu_x, \sigma_x) = \frac{V_{\mathrm{reset}} - \mu_x}{\sigma_x} \tag{S.22}$$

A set of first-order differential equations (Eq. S.23) whose fixed points correspond to solutions of Eq. S.19 are used to solve the entire set of equations.

$$\tau_x \frac{d\nu_x}{dt} = -\nu_x + \Phi(\mu_x, \sigma_x) \tag{S.23}$$

The network dynamics could converge to different attractors states corresponding to multi-item memory states or m-memory states, with m being the number of coactivated subpopulations. The working point was selected based on a systematic scan of the parameter space $\{\omega_I, \omega_+\}$ with the aim to reproduce the experimental data reported in the literature. To this end, a set of initial conditions obtained by varying the number of initially coactivated populations both in the saliency and no saliency condition were considered (see Fig. 2). In order to exhaustively search the possible fixed points for the selected working point $\{\omega_I = 1.15, \omega_+ = 2.2\}$, we integrated Eq. S.23 with 1000 different initial conditions (selected randomly except from those already included in the previous study) for the population firing rates. The same upper working memory capacity limit (i.e. 4 items) found in Fig. 2 was obtained in this study.

References

1. Brunel N, Wang XJ (2001) Effects of neuromodulation in a cortical network model of object working memory dominated by recurrent inhibition. J Comput Neurosci 11: 63-85.
